# Engagement of monocytes, NK cells, and CD4^+^ Th1 cells by ALVAC-SIV vaccination results in a decreased risk of SIV_mac251_ vaginal acquisition

**DOI:** 10.1371/journal.ppat.1008377

**Published:** 2020-03-12

**Authors:** Giacomo Gorini, Slim Fourati, Monica Vaccari, Mohammad Arif Rahman, Shari N. Gordon, Dallas R. Brown, Lynn Law, Jean Chang, Richard Green, Fredrik Barrenäs, Namal P. M. Liyanage, Melvin N. Doster, Luca Schifanella, Massimiliano Bissa, Isabela Silva de Castro, Robyn Washington-Parks, Veronica Galli, Deborah H. Fuller, Sampa Santra, Michael Agy, Ranajit Pal, Robert E. Palermo, Georgia D. Tomaras, Xiaoying Shen, Celia C. LaBranche, David C. Montefiori, David J. Venzon, Hung V. Trinh, Mangala Rao, Michael Gale, Rafick P. Sekaly, Genoveffa Franchini

**Affiliations:** 1 Animal Models and Retroviral Vaccines Section, National Cancer Institute, Bethesda, Maryland, United States of America; 2 Department of Pathology, Case Western Reserve University, Cleveland, Ohio, United States of America; 3 Department of Infectious Diseases, GlaxoSmithKline R&D, Research Triangle Park, North Carolina, United States of America; 4 Department of Immunology, Center for Innate Immunity and Immune Disease, and Washington National Primate Research Center, University of Washington, Seattle, Washington, United States of America; 5 Beth Israel Deaconess Medical Center, Boston, Massachusetts, United States of America; 6 Division of Surgical Sciences, Duke University School of Medicine, Durham, North Carolina, United States of America; 7 Advanced Bioscience Laboratories, Rockville, Maryland, United States of America; 8 Biostatistics and Data Management Section, National Cancer Institute, Bethesda, Maryland, United States of America; 9 U.S. Military HIV Research Program, Walter Reed Army Institute of Research, Silver Spring, Maryland, United States of America; University of Wisconsin, UNITED STATES

## Abstract

The recombinant Canarypox ALVAC-HIV/gp120/alum vaccine regimen was the first to significantly decrease the risk of HIV acquisition in humans, with equal effectiveness in both males and females. Similarly, an equivalent SIV-based ALVAC vaccine regimen decreased the risk of virus acquisition in Indian rhesus macaques of both sexes following intrarectal exposure to low doses of SIV_mac251_. Here, we demonstrate that the ALVAC-SIV/gp120/alum vaccine is also efficacious in female Chinese rhesus macaques following intravaginal exposure to low doses of SIV_mac251_ and we confirm that CD14^+^ classical monocytes are a strong correlate of decreased risk of virus acquisition. Furthermore, we demonstrate that the frequency of CD14^+^ cells and/or their gene expression correlates with blood Type 1 CD4^+^ T helper cells, α_4_β_7_^+^ plasmablasts, and vaginal cytocidal NKG2A^+^ cells. To better understand the correlate of protection, we contrasted the ALVAC-SIV vaccine with a NYVAC-based SIV/gp120 regimen that used the identical immunogen. We found that NYVAC-SIV induced higher immune activation via CD4^+^Ki67^+^CD38^+^ and CD4^+^Ki67^+^α_4_β_7_^+^ T cells, higher SIV envelope-specific IFN-γ producing cells, equivalent ADCC, and did not decrease the risk of SIV_mac251_ acquisition. Using the systems biology approach, we demonstrate that specific expression profiles of plasmablasts, NKG2A^+^ cells, and monocytes elicited by the ALVAC-based regimen correlated with decreased risk of virus acquisition.

## Author summary

The ALVAC-HIV/gp120/alum regimen tested in 8,197 human volunteers (61.4% males, 38.6% females) in the RV144 trial decreased the risk of HIV infection similarly in both sexes. The ALVAC-SIV/gp120/alum vaccine also reduced the risk of intrarectal SIV_mac251_ acquisition in both female and male vaccinated macaques at an average of 44% per exposure. In the current work, we tested whether this vaccine modality could also reduce the risk of intravaginal SIV_mac251_ exposure. In order to detect correlates of risk, we administered the virus by the intravaginal route and tested another vaccine regimen based on the vaccinia derivative poxvirus NYVAC in parallel. We demonstrate here that the ALVAC-SIV/gp120/alum regimen decreases the risk of vaginal SIV_mac251_ acquisition (50% vaccine efficacy) and, importantly, we confirmed that subsets of monocytes and CD4^+^ T cells are correlates of risk of acquisition. In addition, we uncovered cytotoxic vaginal NKG2A^+^ cells and gut-homing α_4_β_7_ positive plasmablasts as novel correlates of risk of intravaginal virus acquisition. In contrast, NYVAC-SIV vaccination induced high levels of activated T cells and did not protect against SIV_mac251_ acquisition. We examined the contrasting immune responses to better understand the correlate of protection and found that the unique ability of ALVAC-SIV to activate early interferon responses and the inflammasome during priming differentiates the two poxvirus vectors. This work demonstrates the reproducibility of the efficacy observed in the ALVAC-based regimen and defines novel correlates of risk in the rigorous SIV_mac251_ macaque model, establishing a benchmark for future improvement of this vaccine approach.

## Introduction

Important advances have been made toward the development of a preventive HIV-1 vaccine, but further work is needed to address this global priority. Most notably, the RV144 HIV vaccine trial tested a recombinant Canarypox ALVAC-HIV vCP1521 vaccine administered in combination with the AIDSVAX B/E vaccine containing two monomeric clade B and AE HIV-1 gp120 proteins formulated in alum, a regimen found to decrease the risk of HIV acquisition by 31.2% [1, 2]. The ALVAC vector is derived from repeated passages of Canarypox in chicken embryo fibroblasts and demonstrated a high level of safety and tolerability in phase I clinical trials in infants [3, 4] and adults [5]. The results of RV144 provided valuable insight into the functionality of the ALVAC vector and revealed that the decreased risk of HIV acquisition correlated with the high serum level of IgG that recognized variable regions 1 and 2 (V1/V2) of the HIV gp120 envelope protein [6]. Serum envelope-specific IgA, in contrast, correlated with an increased risk of viral infection [7, 8]. The presence of ADCC was shown to correlate with a decreased risk of virus acquisition in the presence of low level IgA to gp120 [7]. Notably, only 19.7% of the volunteers enrolled in this trial were found to be positive for CD8^+^ CTL responses as measured by IFN-γ ELISpot, consistent with the lack of viral replication control in the vaccinees that became infected [1]. Conversely, most vaccinees developed polyfunctional Env-specific CD4^**+**^ T cell responses, a secondary correlate of risk of HIV-1 acquisition [9].

The ALVAC-HIV-2 and NYVAC-HIV-2 vaccines afforded protection from the weakly pathogenic HIV-2 in high dose intravaginal (IV) or mucosal challenges in early, preclinical studies in macaques [2, 10]. However, a combined vaccine priming of ALVAC-SIV/Gag-Pol and ALVAC-HIV-1/Env boosted with HIV-1/gp120 did not protect rhesus macaques from acquisition of the chimeric Simian-Human Immunodeficiency Virus ku2 (SHIV_ku2_) following a high dose mucosal challenge, though it did limit T cell loss [11]. Similarly, this vaccine modality did not protect against a high dose mucosal challenge of SIV_mac251_ in adult macaques, and it only transiently reduced plasma virus and CD4^+^ T cell loss in the vaccinees that became infected [12]. The importance of the challenge dose in the efficacy of ALVAC-SIV based regimens against SIV_mac251_ was clearly demonstrated by comparing a single high dose to repeated intermediate intrarectal doses of the virus, and by showing that 10 out of 16 animals immunized with ALVAC-SIV/Gag-Pol-Env remained uninfected after oral repeated exposure to low doses of SIV_mac251_ [13, 14]. More recently, the efficacy of the ALVAC-SIV/gp120 regimen was tested against rectal low dose SIV_mac251_ challenges that infected approximately one third of the control macaques at each challenge while transmitting few virus variants, as notably is the case with HIV in humans. In a comparison of the per-exposure rate of mucosal virus acquisition in vaccinated animals versus controls, this regimen reduced the risk of virus acquisition by 44% [15]. Furthermore, the efficacy of this regimen was modestly augmented to 52% when using a DNA prime [16].

Though the results of RV144 and these macaque studies demonstrate that the ALVAC based vaccines afford some degree of efficacy in humans and macaques, this approach clearly requires improvement. As a possible alternative to ALVAC, we evaluated the vaccinia virus-derived NYVAC vector, attenuated through the deletion of 18 genes that regulate virus host range and virulence [17]. NYVAC is an attractive candidate for this purpose as it undergoes an abortive replication in most mammalian cells, is immunogenic, and it demonstrated an overall good safety profile in phase I studies [18]. In rhesus macaques, NYVAC-HIV-2 provided protection from intravenous exposure to the nonpathogenic HIV-2 strain SBL669 [19], and the inclusion of an Env protein boost in the vaccination protocol further increased protection [20, 21]. In models of exposure to high doses of SIV_mac251_, NYVAC-SIV alone or in combination with the IL-2 and IL-12 cytokines did not protect from virus acquisition, but it delayed disease progression in one-third of vaccinated animals following a single high dose of SIV_mac251_ [22]. Notably, the vaccine effect demonstrated some degree of durability in this study in animals challenged six months after the last immunization [23]. In the same high dose mucosal challenge SIV_mac251_ model, the substitution of the NYVAC-SIV prime with a DNA-SIV did not protect against SIV_mac251_ acquisition, but increased cytotoxic and helper T cell responses that correlated with protection against disease development [23, 24].

In the present study, we tested adult female Chinese rhesus macaques to investigate whether the NYVAC-SIV prime/gp120 regimen could decrease the risk of virus acquisition following exposure to repeated low doses of SIV_mac251_ by the vaginal route. In parallel, we tested the recombinant ALVAC-SIV prime/gp120 boost, since this vaccine modality decreased the risk of HIV acquisition in humans and of SIV_mac251_ acquisition in macaques following intrarectal exposure [1, 15] but was not previously tested against vaginal challenge. Here, we demonstrated that the ALVAC-SIV/gp120/alum vaccine regimen decreases the risk of vaginal SIV_mac251_ acquisition in Chinese rhesus macaques and confirmed that higher levels of classical monocytes correlate with a decreased risk of virus acquisition. Our data further suggest that this monocyte subset affects the frequency and function of NKG2A^+^ cells in vaginal mucosa. Surprisingly, the NYVAC-based vaccine regimen did not decrease the risk of SIV_mac251_ acquisition. This vaccine regimen increased the frequency of activated cells, and systems biology analyses show that NYVAC-SIV elicited a different inflammatory profile than the ALVAC-based regimen. All together, these findings highlight a complex interplay between vaccine-induced innate and adaptive immunity in shaping responses to inhibit SIV acquisition. Noting their differences, we contrast the immune responses elicited by these regimens to better understand the correlate of protection.

## Results

### ALVAC-SIV/gp120 but not NYVAC-SIV/gp120 vaccination reduces the risk of SIV_mac251_ acquisition

**Fig 1:**
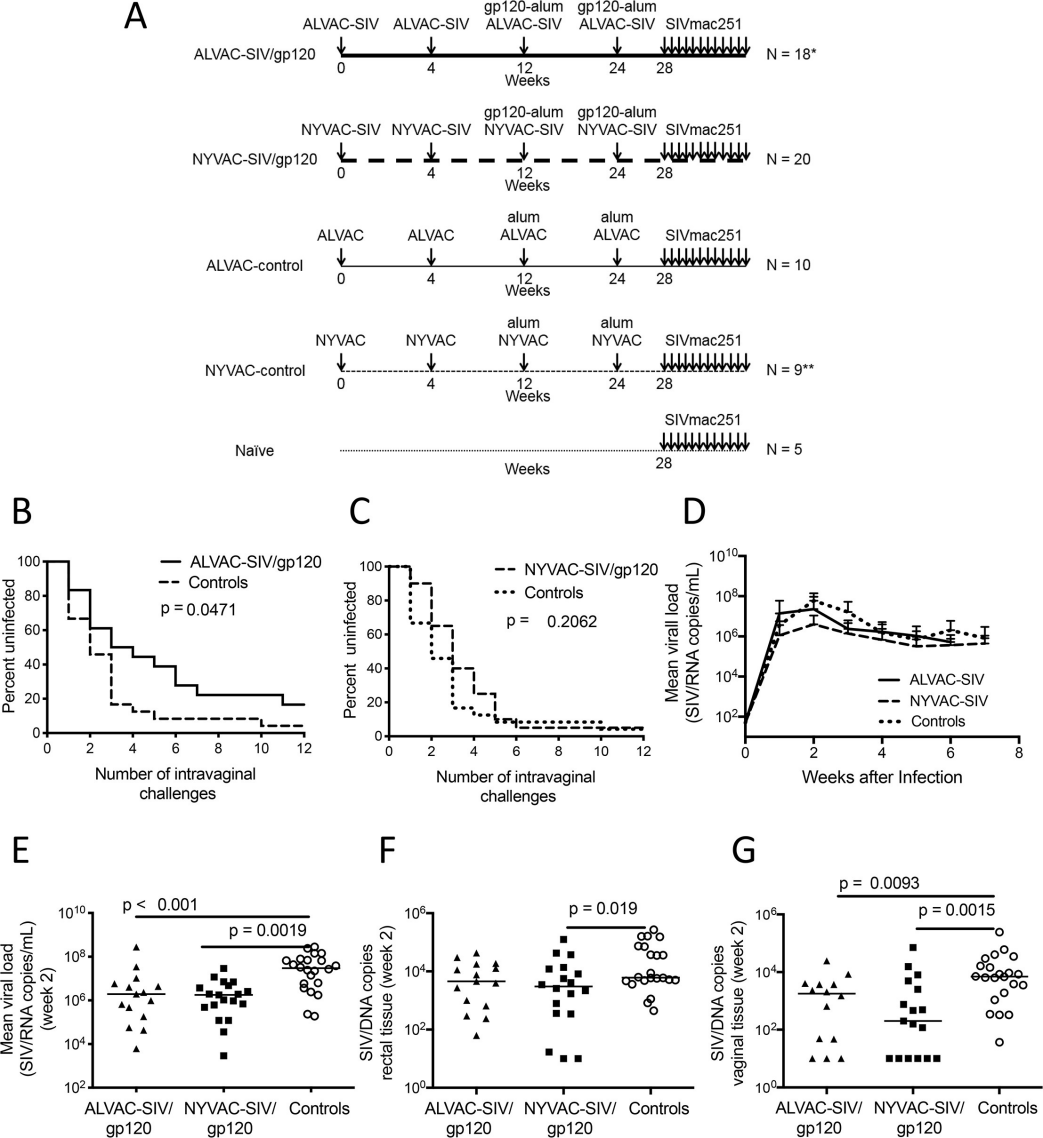
Study design and vaccine efficacy. (A) Study design. Animals were immunized with either ALVAC-SIV (vCP180; 20 animals) or NYVAC-SIV (VP1071; 20 animals) expressing Gag-Pol-Env of the SIV_K6W_ clone of SIV_mac251_ [20] and boosted with the native form of SIV_mac251_/gp120 adjuvanted in 5 mg of alum Alhydrogel. Two of the 20 animals included in the ALVAC-SIV group and one of the 10 animals in the NYVAC-parental group died before the challenge phase for reasons unrelated to the vaccine. (B, C) Acquisition curves following SIV_mac251_ intravaginal administration in the (B) ALVAC-SIV (n = 18) and (C) NYVAC-SIV immunized (n = 20) animals compared to the pooled control groups (**S1A Fig**). (D) Logarithmic mean ± s.d. of SIV/RNA levels in the plasma of the animals that became infected in the three animal groups (ALVAC-SIV, n = 18; NYVAC-SIV, n = 19; pooled controls n = 20). (E) Logarithmic mean ± s.d. of SIV/RNA levels in the plasma of the same infected animals 2 weeks from infection. SIV/DNA copy number in the (F) rectal and (G) vaginal mucosa of the infected animals amongst the vaccinated (ALVAC-SIV n = 14; NYVAC-SIV n = 17) and control (n = 20) groups 2 weeks post-infection (horizontal line: median).

Forty female Chinese rhesus macaques were distributed equally in the groups based on age, and weight (**Table 1**). Twenty animals were immunized in each vaccine group with either ALVAC-SIV or NYVAC-SIV expressing identical SIV genes (**Fig 1A**). Animals were first immunized at weeks 0 and 4, and boosted at weeks 12 and 24 with the corresponding viral vector in one limb and a single monomeric native SIV_mac251_/gp120 protein formulated in 5 mg of alum in the contralateral limb. A total of 25 macaques were used as controls: two groups of ten macaques each were immunized with the parental viral vectors and adjuvant, while five animals were left naïve(**Fig 1A**). The number of animals used in each immunization group was not sufficient to compare the relative efficacy of the ALVAC-SIV/gp120 (ALVAC-SIV) and NYVAC-SIV/gp120 (NYVAC-SIV) based vaccines. Rather, this population was adequate to compare each vaccine to mock vaccinated or naïve controls. Four weeks following the last immunization, twelve consecutive challenges were performed with a weekly low intravaginal dose of SIV_mac251_ using a challenge stock with high genetic diversity, propagated in macaque cells by R. C. Desrosiers [25]. The study was divided into two parts (Part 1 and Part 2) and conducted in two separate animal facilities (**Table 1**). Before the initiation of the study, it was decided that if no difference in the rate of SIV_mac251_ acquisition was observed among the three control groups, vaccine efficacy would be assessed by pooling all controls, and comparing them the vaccinees immunized with each vaccine regimen. The control groups (ALVAC-control, NYVAC-control, and Naïve groups) did not differ significantly from each other following intravaginal challenge exposure to SIV_mac251_ (**S1A Fig**). Additional data showing that the ALVAC-SIV group had an estimated vaccine efficacy of 50% at each challenge (Log-rank test: *p* = 0.0471; **Fig 1B**) confirmed and built upon the results of one of our prior studies of an equivalent vaccine regimen tested against intrarectal challenges in male and female Indian rhesus macaques [15]. Unexpectedly, vaccination with NYVAC-SIV did not decrease the risk of virus acquisition in vaccinated animals (Log-rank test: *p =* 0.2062; **Fig 1C**). While there was no overall difference in the plasma virus levels of the vaccinated animals that became infected and the controls (**Fig 1D**), a transient reduction of viremia was observed in the NYVAC group (2 weeks from infection; NYVAC vs. pooled controls *p* = 0.0019) and in the ALVAC-group (ALVAC vs. control *p* < 0.001, by the Wilcoxon-Mann-Whitney test, not corrected for multiple comparisons; **Fig 1E**). To determine whether vaccination affected virus levels in mucosal tissues, we quantified SIV DNA copies in the rectal and vaginal biopsies at 2 weeks post-infection. In rectal tissue, we found a significant difference only between the NYVAC-SIV and control groups (*p =* 0.019; **Fig 1F**), whereas both ALVAC-SIV and NYVAC-SIV immunized animals had significantly lower SIV DNA copies than the control group in the vaginal mucosa (*p =* 0.0093 and *p* = 0.0015, respectively; **Fig 1G**).

**Table 1:** Vaccination groups.

Group	Facility		Total
	Part 1	Part 2	
**ALVAC-SIV/gp120**	7^a^	11^a^	18
**NYVAC-SIV/gp120**	8	12	20
**ALVAC-control**	4	6	10
**NYVAC-control**	3^a^	6	9
**Naïve**	n/a	5	5
**Total**	22	40	**62**

Of the 65 animals, 62 underwent the challenge phase.

^a^Groups in which a macaque was sacrificed before challenge due to complications from self-inflicted wounds unrelated to the vaccine.

### Monocyte subsets and MDSCs differently affect the risk of vaginal SIV_mac251_ acquisition in the ALVAC-SIV regimen

**Fig 2:**
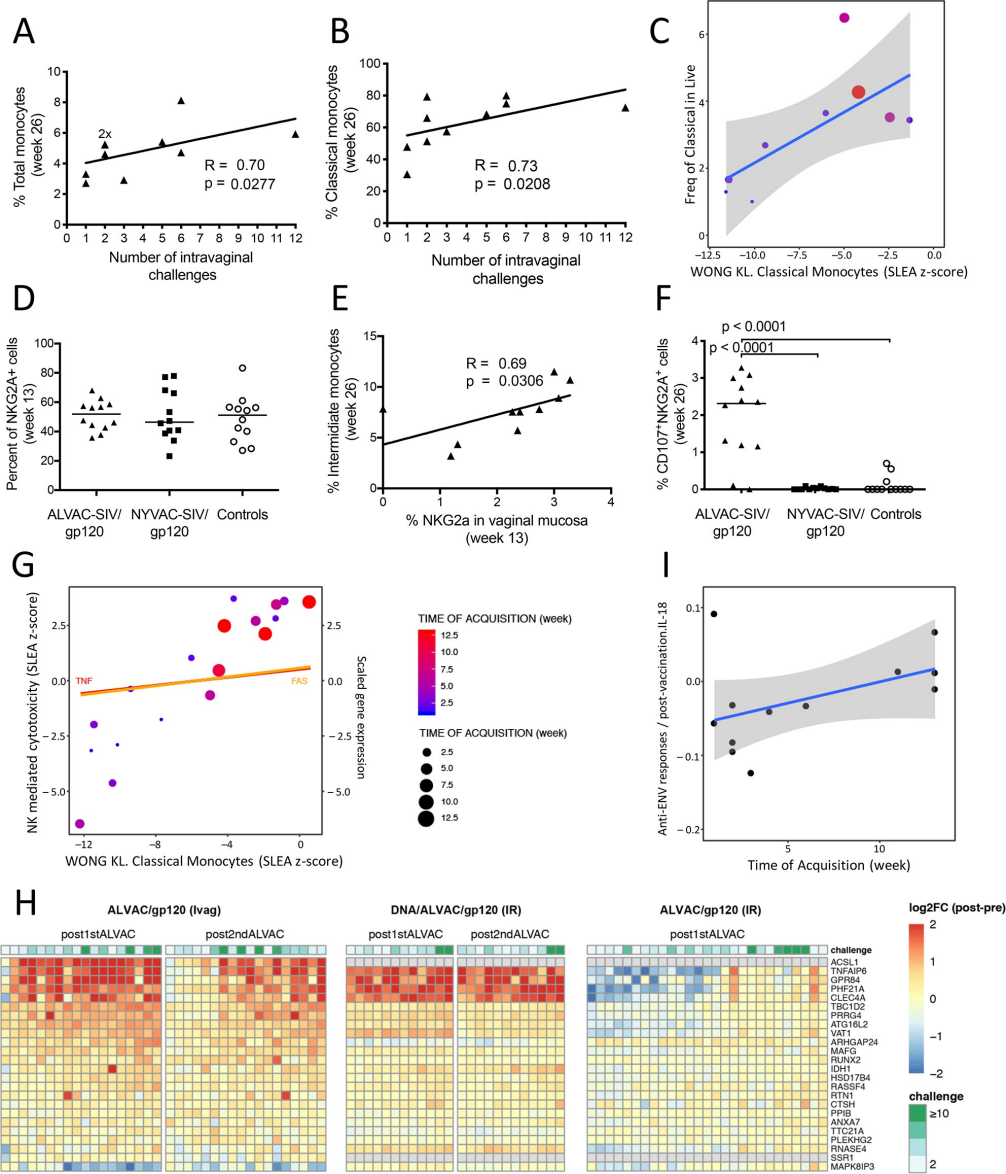
Monocytes and NKG2A cells in risk of SIV_mac251_ acquisition. Pearson correlation of myeloid cell subsets with SIV_mac251_ acquisition in the ALVAC-SIV group. The levels of myeloid cells were analyzed only from animals in Part 2 (Table **1**). (A) The percentage of total monocytes and (B) CD14^+^CD16^-^ classical monocytes were associated with decreased rate of SIV acquisition. (C) GSEA analysis of the transcriptomic profile of the ALVAC-SIV/gp120 animals at week 26 revealed an enrichment of classical monocyte markers among genes associated with lower risk of SIV acquisition. The SLEA method was used to summarize classical monocyte markers for each subject. A scatterplot shows average classical monocyte markers as a function of the frequency of classical monocytes measured by FCM in ALVAC-SIV treated animals at week 26. The grey region indicates the 95% confidence-interval of this correlation. (D) Relative frequency of NKG2A cells (defined as CD45^+^, CD3^-^, CD20^-^, and CD14^-^ cells) at week 13 for the ALVAC-SIV/gp120, NYVAC-SIV/gp120, and control groups from Part 2 (horizontal line: mean). (E) Correlation between the frequency of intermediate monocytes in blood at week 26 and the cytotoxic function of mucosal NKG2A cells (week 13). (F) Frequency of vaginal NKG2A CD107^+^ cells in the ALVAC-SIV vaccinated, NYVAC-SIV vaccinated, and control groups from Part 2 of the study at week 26 (horizontal line: mean). (G) Scatterplot of the average of cytotoxic NK cell markers as a function of the average expression of classical monocyte markers. The gene expression of FAS and TNF, two canonical cytotoxic NKs, as function of the expression of classical monocyte markers is indicated by lines. (H) Heatmaps showing the level of expression of transcriptomic markers of classical monocytes in three NHP studies. In the current study (ALVAC/gp120 [Ivag]; left), animals were primed with ALVAC-SIV and boosted with ALVAC-SIV+gp120 formulated in alum. Blood samples were taken 24h after the first boost (week 12) and after the 2nd boost (week 24). In a prior study (DNA/ALVAC/gp120 [IR]; center), animals were primed with DNA and boosted with ALVAC-SIV/gp120 formulated in alum [16]. Blood samples were taken 24h after the first (week 12) and second (week 24) boosts. In another of our prior studies (ALVAC/gp120 [IR]; right), animals were primed with ALVAC-SIV, boosted with ALVAC-SIV+gp120 formulated in alum, and challenged intrarectally [15]. Blood samples were taken 24 h after the first ALVAC-SIV immunization. GSEA enrichment analysis was used to test for enrichment of transcriptomic markers of classical monocytes [80] among genes correlated with challenges in each study (ALVAC/gp120 [Ivag]: 1st boost, NES = 2.70, FDR < 0.001; 2nd boost, NES = 2.33, FDR < 0.001; DNA/ALVAC/gp120 [IR]: 1st boost: NES = 1.23, FDR = 0.104; 2nd boost: NES = 0.888, FDR = 0.779; ALVAC-SIV/g120 [IR]: NES = 1.54, FDR = 0.146). The markers of classical monocytes correlating with challenges (i.e. leading edge genes) overlapping between the three studies are shown in the heatmap. A blue-to-red color gradient represents the log2 fold-change between post-vaccination and pre-vaccination gene expression. A Spearman correlation and t-test were used. The Spearman correlations were transformed to t statistics and compared to the Student distribution (t-test) using the formula below.
$$t*=rho\times \frac{\sqrt{n-2}}{\sqrt{1-{rho}^{2}}}$$
In this formula, rho is Spearman’s rho for samples, t is taken to be (1 –[a/2]) of the t-distribution (with n– 2 degrees of freedom), and null hypothesis significance testing rejects the null if |t*| is greater than or equal to t. This was done to statistically assess the correlation between the ordering of the samples by the levels of gene expression and challenge (ALVAC/gp120 [Ivag]: 1st boost, R = 0.48, *p* = 0.0433; 2nd boost, R = 0.15, *p* = 0.5464; DNA/ALVAC/gp120 [IR]: 1st boost, R = 0.71, *p* = 0.0089; 2nd boost, R = 0.37, *p =* 0.2411; ALVAC-SIV/gp120 [IR]: R = 0.30, *p* = 0.1305). (I) Scatterplot of average IL18 measured by Luminex assay following envelope stimulation of blood cells at week 13 as a function of the number of SIV challenges to infection in the ALVAC-SIV/gp120 treated animals. The grey region indicates the 95% confidence-interval of this correlation.

CD14^+^ classical, CD14^+^CD16^+^ intermediate, and CD14^-^CD16^+^ non-classical monocytes and myeloid derived suppressor cells (MDSCs) play a key role in the modulation of adaptive CD4^+^ and CD8^+^ T and B cell responses induced by poxvirus vectors [26] and HIV and SIV infection [27–29]. Measurement of the frequency of each monocyte subset in blood before immunization (week 0) and two weeks following the final immunization (week 26) revealed that the two vaccine regimens did not significantly change the overall blood frequency of total or classical monocytes (**S1B and S1C Fig**), nor of CXCR4^+^CD14^+^CD16^-^ classical monocytes (**S1D Fig**). Similarly, the frequency of total, CXCR4^+^, intermediate CD14^+^CD16^+^, or non-classical CD14^-^CD16^+^ monocytes did not differ between the groups (**S1E–S1H Fig**). Analysis of the frequency of CCR2^+^ monocyte subsets only demonstrated a significantly higher frequency (*p* = 0.0269) of the CCR2^+^CD14^+^CD16^+^ intermediate monocyte subset in the macaques immunized with NYVAC-SIV (**S1I, S1J and S1K Fig**). Notably, the frequency of both total and classical monocytes in the ALVAC-SIV group correlated positively with delayed virus acquisition (R = 0.70, *p* = 0.0277 and R = 0.73, *p* = 0.0208, respectively; **Fig 2A and 2B**), confirming our prior results with the ALVAC-based vaccine modality [16]. In contrast, we did not find a correlation between CXCR4^+^ monocytes and the risk of virus acquisition in the present study. The different vaccine regimens and sample collection times between the current (week 26, ALVAC-SIV prime) and prior (week 27, DNA prime) studies may possibly account for this difference.

In agreement with our prior work, changes in gene expression in the blood of animals in the ALVAC-SIV group (week 26 compared to pre-vaccination) supported the association of classical monocyte cells and inflammasome activation with decreased risk of SIV acquisition (**Fig 2C**). While no association was observed between SIV acquisition and monocyte frequency in the NYVAC-SIV group, classical monocyte genes associated with the decreased risk conferred by ALVAC-SIV vaccination included *AKR1B1* (a marker of M1 macrophage polarization [30]), *CCL3* and *CDKN1A* (monocytic inhibitors of HIV-1 replication [31, 32]), *CCR2* and *CD44* (promotors of monocyte chemotaxis [33, 34]), *CD14* (the co-receptor of LPS at the surface of monocytes), *CLEC7A* (an inducer of NLRP3 inflammasome [35]), *IL1β* (the byproduct of NLRP3 inflammasome activation [36]) and its receptor *IL1R2* [37], and *F13A1* (implicated in the phagocytic activities of monocytes [38]). In contrast, the frequency of non-classical CD16^+^ monocytes and CD14^+^ HLA-DR^-^ cells (MDSCs) correlated with earlier virus acquisition as expected (**S1L and S1M Fig**).

### Classical and intermediate monocytes in blood are associated with cytocidal vaginal NKG2A

In a prior study, we found that ALVAC-SIV/gp120 immunization increased the frequency of NKp44^+^ cells in the rectal mucosa that in turn correlated with delayed virus acquisition following rectal exposure to SIV_mac251_ [15]. Furthermore, the frequency of mucosal NKp44^+^ cells correlated with the plasma level of CCL2, a chemokine produced at a high level by classical monocytes [39]. NK cells are considered to be the first line of defense against viral infections [40] because of their ability to exert cytotoxic activity toward virus-infected cells without the need for MHC-mediated activation, and their regulation of the inflammatory milieu. Consistent with prior reports, we observed low level NKp44^+^ cells and the prevalence of NKG2A^+^ cell in vaginal mucosa [41, 42]. The frequency of NKG2A^+^ cells (defined as CD45^+^ CD3^-^ CD20^-^ CD14^-^ NKG2A^+^ cells) in vaginal mucosa did not differ among vaccinated and control animals at week 13 (**Fig 2D** and **S2A Fig**). The frequency of vaginal NKG2A^+^ cells correlated with the level of intermediate monocytes at the end of immunization (R = 0.69, *p* = 0.0306, week 26; **Fig 2E**). Vaginal NKG2A^+^ cells with cytotoxic profiles (CD107a^+^) were significantly higher in ALVAC-SIV immunized macaques than in the NYVAC-SIV or control groups (*p* < 0.0001; **Fig 2F**). Interestingly, gene expression related to NK cytotoxicity directly correlated with the classical monocyte transcriptomic signature and decreased risk of SIV_mac251_ acquisition (**Fig 2G**). CD14^+^ monocytes are known to recruit cytotoxic NK cells following inflammasome activation and production of IL-18. We found that cells stimulated with envelope overlapping peptides produced a cytokine profile marked by a trend of IL-18 and the average gene expression of classical monocyte markers (Spearman correlation: R = 0.35, *p* = 0.266; **Fig 2H**). IL-18 also trended with the number of SIV challenges to infection (Spearman correlation: R = 0.46, *p* = 0.1311; **Fig 2I**). Although these individual correlations are weak, together they support the hypothesis that ALVAC-SIV vaccination engages CD14^+^ monocytes and affects the function of cytocidal vaginal NKG2A^+^ cells, via IL-18 production.

**Fig 3:**
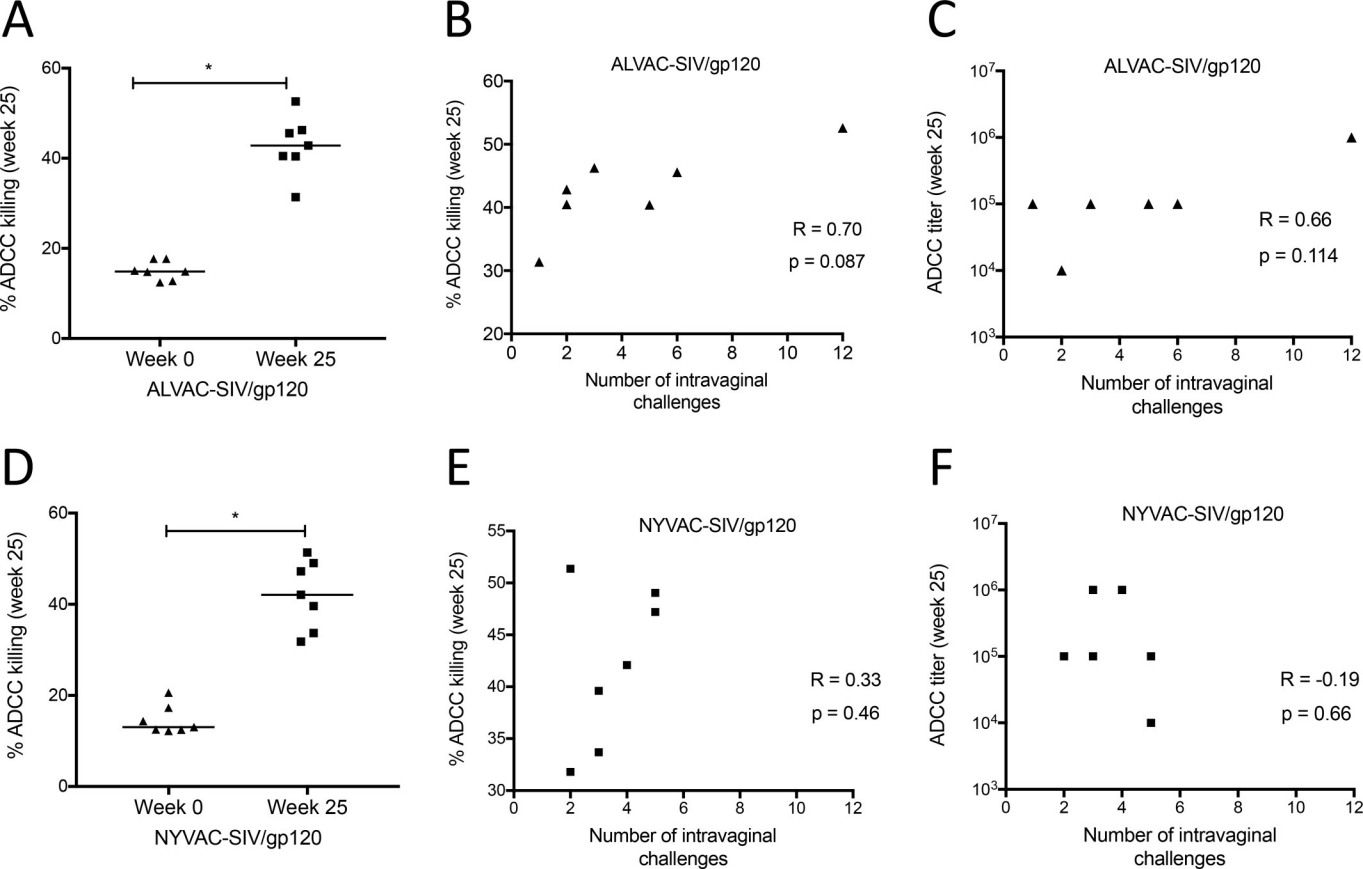
ADCC activity in ALVAC-SIV/gp120 and NYVAC-SIV/gp120 vaccinated animals. Comparison of percent ADCC killing between pre-immunization and one week post-final immunization (week 25) in (A) 7 ALVAC and (D) 7 NYVAC-vaccinated macaques. (B,C) A positive correlation was observed between the number of intravaginal challenges and the percentage of ADCC killing or ADCC titers in ALVAC-vaccinated macaques. (E,F) Correlation analysis between the number of intravaginal challenges to infection and the percentage of ADCC killing or ADCC titers in 7 NYVAC-vaccinated macaques. Horizontal lines represent the median.

To further investigate functional cytocidal cells in blood, we measured ADCC activity and ADCC titers mediated by plasma from 7 animals from each group in Part 2 of the study using purified SIV_766_ gp120 coated target cells [43]. ADCC activity was measured in these ALVAC-SIV and NYVAC-SIV vaccinated animals one week after the last immunization (**Fig 3A and 3D**). The ADCC titers did not differ at the end of immunization in the two groups (**S2B and S2C Fig**). There was, however, a trend between both ADCC activity and ADCC titers with delayed SIV_mac251_ acquisition in the ALVAC-SIV group (**Fig 3B and 3C**), but not in the NYVAC-SIV group (**Fig 3E and 3F**).

### Vaccine-induced CD4^+^ Th1 cells correlate with CD14^+^ monocytes and decreased risk of SIV_mac251_ acquisition

**Fig 4:**
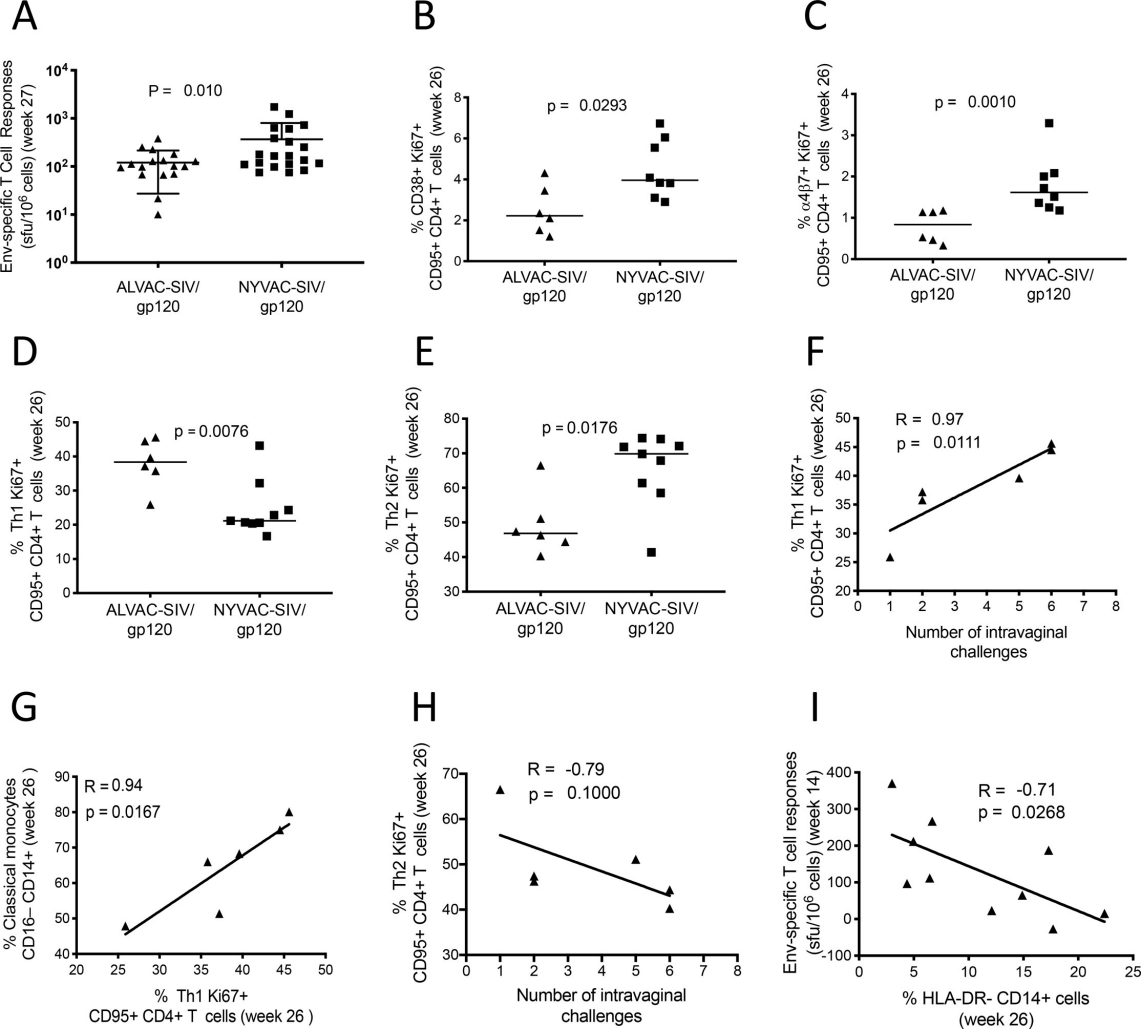
CD4^+^ T cell subsets. (A) T cell ELISpot in the vaccinated group at 3 weeks after the last immunization (week 27). CD4^+^ T cells were measured in 6 ALVAC-vaccinated and 8 NYVAC-vaccinated macaques. (B) Percentage of vaccine-induced (Ki67) CD38^+^CD4^+^ T cells at week 26. (C) Percentage of KI67^+^ α4β7^+^CD4^+^ T cells at week 26. (D) Frequency of Th1 (CXCR3^+^ CCR6^–^) and (E) Th2 (CXCR3^–^ CCR6^–^) CD4^+^ T cells at week 26 (horizontal line: median). (F) Direct associations between the levels of ALVAC-SIV/gp120 induced Th1 cells at week 26 and the number of challenges to infection or (G) to the frequency of classical monocytes in blood at week 26. (H) Inverse correlation of Th2 cells measured at week 26 and time of SIV_mac251_ acquisition. (I) Correlation of CD14^+^HLA-DR^-^ (MDSCs) measured at week 14 and the level of anti-envelope ELISpot responses in blood at week 14.

The NYVAC-SIV and ALVAC-SIV vaccines induced equivalent SIV-Gag IFN-γ ELISpot responses (**S2D Fig**), whereas SIV-envelope IFN-γ ELISpot responses at the end of each regimen were significantly higher in the NYVAC-SIV group (*p* = 0.010; **Fig 4A** and **S2E Fig**). Two weeks following the last immunization (week 26), animals vaccinated with NYVAC-SIV had a significantly higher percentage of vaccine-induced (Ki67^+^) activated CD38^+^ and gut-homing α_4_β_7_^+^ CD4^+^ T cells in blood than did the ALVAC-vaccinated animals (*p* = 0.0293 and *p* = 0.0010, respectively, Mann-Whitney test; **Fig 4B and 4C**). In contrast, macaques vaccinated with ALVAC-SIV had significantly higher vaccine-induced Ki67^+^CXCR3^+^CCR6^-^ Th1-type CD4^+^ T cells (*p* = 0.0076; **Fig 4D** and **S2F Fig**) and significantly lower total CXCR3^-^CCR6^-^ Th2-type CD4^+^ T cells (*p* = 0.0176; **Fig 4E**) in blood than the NYVAC-vaccinated animals. The frequency of Th1 CD4^+^ cells in ALVAC-vaccinated animals was strongly associated with both decreased risk of SIV_mac251_ acquisition (R = 0.97, *p* = 0.0111; **Fig 4F**) and the level of classical monocytes (R = 0.94, *p* = 0.0167; **Fig 4G**). The levels of CCR5 on CD4^+^ T cells were similar in both vaccines (**S2G Fig**). Conversely, there was a negative trend between the frequency of CD4^+^ Th2 cells and early SIV_mac251_ acquisition in the same group (R = -0.79; *p* = 0.100; **Fig 4H**). Together, these data demonstrate that ALVAC and NYVAC vectored vaccines induce functionally different CD4^+^ T cell subsets and that classical monocytes play a key role in the induction of CD4^+^ Th1 cells associated with reduced risk of SIV_mac251_ acquisition. The importance of CD4^+^ Th1 cells is further supported by the finding that the frequency of CD14^+^ HLA-DR^-^ cells (MDSCs), whose frequency is associated with an increased risk of virus acquisition (**S1M Fig**), is also associated with the suppression of envelope-specific Th1 CD4^+^ IFN-γ producing cells (R = -0.71, *p* = 0.0268; **Fig 4I**). No differences were found in the percentage of other phenotypically defined CD4^+^ T cell subsets, such as Th17 and T follicular helper cells, nor was a correlation found between any of the CD4^+^ T cell subtypes and the rate of SIV_mac251_ acquisition in the animals immunized with NYVAC.

### Frequency of gut-homing α_4_β_7_^+^ plasmablasts correlates with decreased risk of SIV_mac251_ acquisition

**Fig 5:**
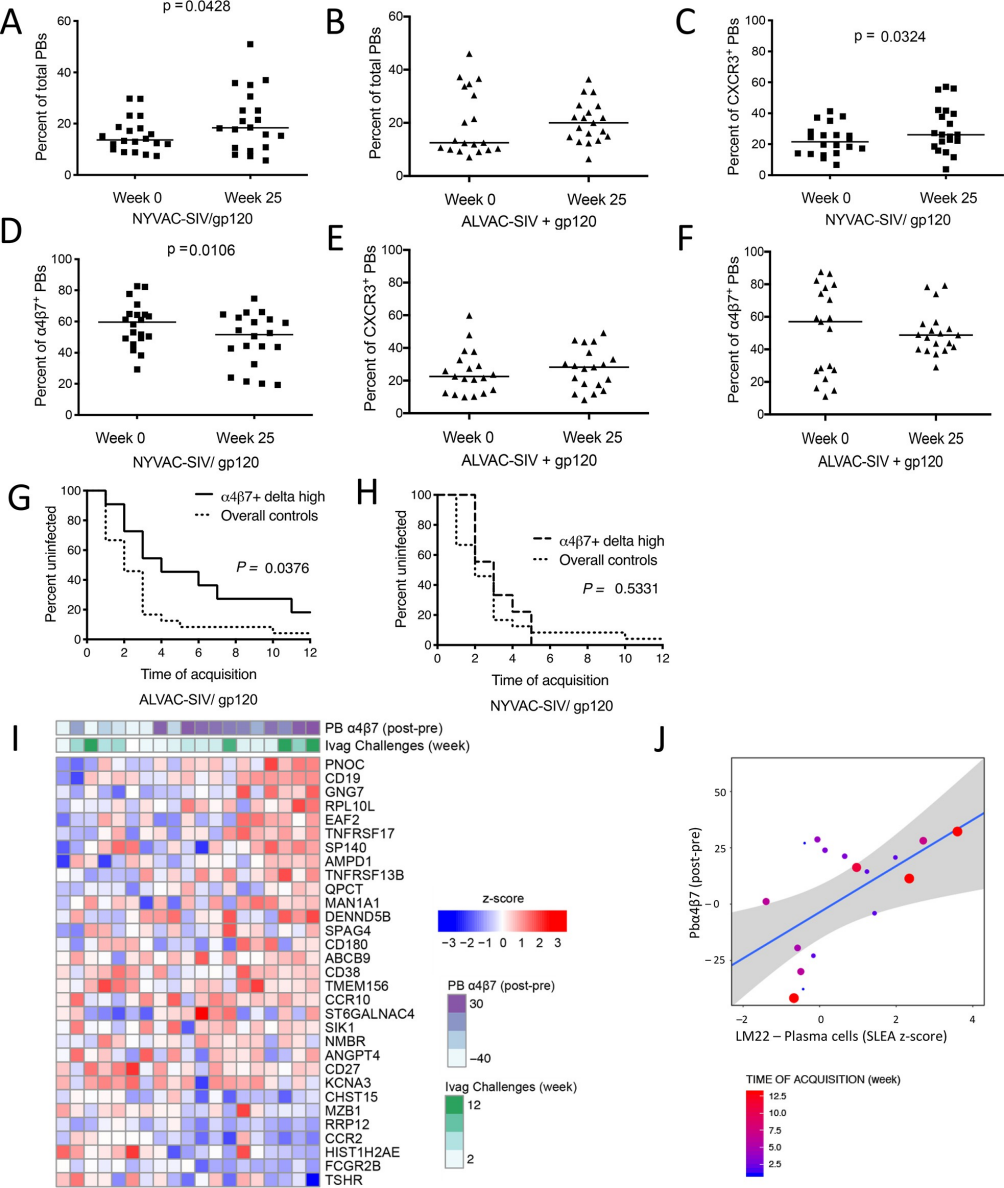
Vaccine-induced plasmablasts and risk of SIV_mac251_ acquisition. Comparison of the vaccine-induced variations in the frequency of plasmablasts (PBs) at week 0 and week 25. Total PBs in (A) NYVAC-SIV and (B) ALVAC-SIV groups. Percent of (C) CXCR3^+^, and (D) α4β7^+^ PBs in the NYVAC-SIV group. Percent of (E) CXCR3^+^ and (F) α4β7^+^ PBs in the ALVAC-SIV group. (G–H) Survival curve showing significant protection in animals with above average increases in levels of α4β7^+^ PBs (from baseline levels) and time to acquisition compared to controls in the (G) ALVAC-group and (H) in the NYVAC group. (I) GSEA analysis of the transcriptomic profile of the ALVAC-SIV/gp120 animals at week 25 revealed an enrichment of plasma cell markers among genes associated with lower risk of SIV acquisition. (J) The SLEA method was used to summarize plasma cell markers for each subject. The scatterplot shows average plasma cell markers as a function of the frequency of α4β7^+^ PBs in ALVAC-SIV/gp120 treated animals at week 25. The size of the dots is proportional to the number of SIV challenges to infection. The grey area indicates the 95% confidence region of this correlation.

To assess the effect of vaccination on B cells, we analyzed blood plasmablasts (PBs) [15] and the α_4_β_7_ and CXCR3 homing markers on the surface of PBs before immunization (week 0) and one week after the last immunization (week 25). The α_4_β_7_ integrin mediates lymphocyte migration to mucosal sites by binding to MAdCAM-1, where it is expressed on the inner surface of the mucosal venules [44]. As a chemokine receptor, CXCR3 binds chemokines CXCL9 and CXCL10, generally released at the site of inflammation where CXCR3-expressing cells are recruited [45].Vaccination with NYVAC-SIV, but not ALVAC-SIV, induced a significant increase in the percentage of total circulating PBs after vaccination (*p* = 0.0428; **Fig 5A and 5B**). In the NYVAC group, the PB level rose due to an increase in CXCR3^+^ plasmablasts (*p* = 0.0324; **Fig 5C**) and a smaller decrease in α_4_β_7_^+^ PBs (*p* = 0.0106; **Fig 5D**).

Significant changes in total, α_4_β_7_^+^, or CXCR3^+^ plasmablasts were not observed in the ALVAC-SIV group (**Fig 5E and 5F**). Analysis of antibody responses to the envelope protein revealed different kinetics of induction of antibodies to gp120 that were faster in NYVAC-SIV, but the level of envelope-specific systemic antibodies or mucosal IgG to the gp70 V1/V2 scaffold did not differ between the two groups at the end of immunization (**S3A and S3B Fig**). The differential pre- and post-vaccination frequencies (delta) of total PBs correlated positively with the level of mucosal IgG to the SIV_mac251_ V1/V2 scaffold in both groups (NYVAC-SIV, R = 0.54, *p* = 0.0296; ALVAC-SIV, R = 0.58, *p* = 0.027). Animals from both groups mounted equivalent neutralizing antibodies to the Tier 1A SIV (**S3C Fig**), but no neutralizing responses were elicited against the Tier 2 virus (**S3D Fig**). However, none of the antibody responses measured above correlated with the risk of SIV_mac251_ acquisition.

Analysis of the differential pre-and post-vaccination levels of α_4_β_7_^+^ PBs (frequencies above the average) correlated with a lower risk of SIV_mac251_ acquisition in ALVAC-SIV vaccinated animals compared to controls (*p* = 0.0376; **Fig 5G**), whereas no differences were observed in NYVAC-SIV immunized animals (*p* = 0.5331; **Fig 5H**). Transcriptomic profiling of PBMCs from ALVAC-SIV/gp120 identified genes (**Fig 5I**) whose expression trended with a decreased risk of virus acquisition (R = 0.43, *p* = 0.0766; **Fig 5J**), including *TNFRSF13B* (a promoter of B cell proliferation and plasma cell differentiation [46]), *CCR10* (encoding a chemokine receptor allowing plasmablast homing to mucosal Ab sites [47]), *MZB1* (a chaperone essential for plasma cell differentiation [48]), *TNFRSF17* (a plasma cell pro-survival gene [49]), CD27 (a marker of mature memory B cells [50]), CD38 (a marker of long-lived plasma cells [51]), and both *FCGR2B* and *CD19* (two inhibitors of B cell differentiation to plasma cells [52]).

### NYVAC and ALVAC based vaccines differently affect gene expression

Data analysis of whole blood obtained following the immunizations revealed that ALVAC-SIV induced a much stronger innate transcriptomic response than NYVAC-SIV. The response primarily occurs early enough that most of the differentially expressed (DE) genes can be identified within 24 hours of immunization (**Table 2; S3E** and **S3F Fig**). Interestingly, gene expression profiles were still altered one week after immunization, even if at a lower degree, and the animals in the ALVAC-SIV group demonstrated the highest overall number of DE genes. ALVAC-SIV specifically induced interferon signaling (**S4 Fig**) that included previously reported antiviral genes (*MX1*, *MX2*, *MYD88*) also induced in the RV144 trial (e.g. *IRF7* in [53]), suggesting that findings from this study may be transposed to human HIV vaccines.

**Table 2:** Differential gene expression following ALVAC-SIV and NYVAC-SIV vaccination.

	ALVAC-SIV vs ALVAC-control (upregulated / downregulated)		NYVAC-SIV vs NYVAC-control (upregulated / downregulated)	
Time From Immunization	DEGs	mean |log2FC|	DEGs	mean |log2FC|
6 h	**0/8**	**1.03**	0/0	
24 h	**5/2**	**1.35**	0/2	1.33
1 week	**4/19**	1.00	0/1	**1.35**
2 weeks	0/1	1.36	**0/2**	**1.43**
4 weeks	**0/1**	**1.13**	0/0	
4 weeks, 6 h	6/4	**1.22**	0/0	
5 weeks	**1/18**	**1.10**	0/0	
12 weeks	0/1	**1.26**	0/1	0.962
12 weeks, 6 h	0/7	**1.45**	**5/11**	0.955
12 weeks, 24 h	**50/39**	0.833	0/7	**1.01**
14 weeks	**1/1**	1.25	0/1	**1.35**
24 weeks, 24h	0/2	**1.17**	2/0	0.900
25 weeks	0/0		**0/2**	**1.40**
26 weeks	**0/5**	**1.35**	0/0	
Total	**67/108**	**1.19**	7/27	1.18

Number of genes differentially expressed (cutoff: LIMMA adj. p ≤ 0.05) and average log fold-change for ALVAC-SIV and NYVAC-SIV compared to their respective controls. The bold number indicates the greatest number of differentially expressed genes (DEGs) between ALVAC-SIV and NYVAC-SIV and the strongest magnitude of differential expression (mean |logFC|) for each timepoint after immunization. ALVAC-SIV induced the greatest number of DEGs and the greatest fold-difference.

## Discussion

Our study demonstrates that the canarypox based ALVAC-SIV/gp120/alum regimen decreases the risk of vaginal acquisition of SIV_mac251_ in Chinese rhesus macaques with a vaccine efficacy of 50%. These results reproduce the efficacy afforded by the ALVAC-SIV/gp120/alum regimen in male and female Indian rhesus macaques (44% efficacy) following intrarectal exposure to the same virus stock [15] and reaffirm the efficacy of this vaccine strategy. In contrast, an identical vaccine regimen vectored with the vaccinia-derivative NYVAC was surprisingly not efficacious. The immune responses induced by these two vaccine regimens were marginally higher in NYVAC-SIV immunized animals as also observed in macaque studies using HIV clade C immunogens [54]. Importantly, similar results were reported from the recently completed HVTN096 HIV trial in humans [55], demonstrating that the NYVAC-HIV vector expressing clade C gp140 in combination with the bivalent heterologous boost used in RV144 (clade B MNgp120 and clade AE A244gp120) elicited 2–4-fold higher antibody responses to fewer V1/V2 scaffolds than in RV144. As in RV144, the antibody response to V1/V2 was not sustained. The modest difference between the antibody responses of RV144 and HVTN096 trials may have also been influenced by the prime, the immunogen expressed in the poxvirus vectors (gp120 in RV144; gp140 in HVTN096), and by the relation of the prime envelope to the boost envelope (homologous in RV144; heterologous in HVTN096). The DNA prime in HVTN906 was also tested in combination with NYVAC-HIV and found to elicit marginally higher CD4^+^ and CD8^+^ T cell responses than the NYVAC-HIV prime, although the responses were not sustained. Similar observations were reported in macaques using a DNA-SIV/NYVAC-SIV vaccine regimen without the gp120 protein boost [24].

By integrating phenotypic cell subset analyses and functional immunological assays with systems biology in the present study, we demonstrate that the ALVAC regime induces a higher level of durable interferon responses during priming than NYVAC-SIV that support the distinct kinetic of induction of cytokines and chemokines within the first 24 hours from immunization [56].

The differences between ALVAC and NYVAC may derive from their different host ranges. The Canarypox-based ALVAC only replicates effectively in avian species [18, 57], and it therefore did not evolve the necessary genetic determinants to hijack or inhibit the more complex immune systems of mammals. In contrast, poxviruses such as the vaccinia derivative NYVAC, smallpox, and monkeypox co-evolved with mammals, allowing for the selection of multiple genes to counteract mammalian immune responses [58]. We demonstrate here that ALVAC is more effective than NYVAC in harnessing innate responses against the heterologous genes expressed by the recombinant ALVAC vectored vaccines. Accordingly, recombinant ALVAC-HIV vaccines have induced high levels of ALVAC-specific CD8^+^ T cell responses in humans, but negligible cytotoxic CD8^+^ T cells to the HIV insert [59]. ALVAC-SIV induces protracted interferon production during priming, as demonstrated by systems biology. Importantly, a strong interferon signature was also observed in RV144 vaccinees [53]. ALVAC (but not NYVAC) possesses the ability to preferentially infect CD14^+^ monocytes [60], activate the inflammasome, and induce the release of both IL-1β and IL-10. Within the first 24 hours of vaccination with ALVAC, the plasma levels of IL-1β and IL-10 increase by 20–50-fold [56]. The finding that NYVAC-SIV immunization did not reduce the risk of SIV_mac251_ acquisition was somehow unexpected given the overall similarity of SIV specific responses elicited by the two vaccine regimens, including antibodies to V1/V2, neutralizing antibody titers, and ADCC.

A more in-depth investigation of vaccine-induced immuneresponses revealed significant qualitative differences in T cell responses and homing markers on plasmablasts. We found that CD4^+^ Th1 cells were higher in the ALVAC-SIV group and correlated with a decreased risk of SIV_mac251_ acquisition. In contrast, CD4^+^ T Th2 cells were higher in the NYVAC-SIV group and correlated with an increased risk of SIV_mac251_ acquisition. Oddly, the CD95^+^Ki67^+^CD4^+^ Th2 cell subset correlated with a decreased risk of SIV_mac265_ acquisition in a study whereby the ALVAC-SIV prime was substituted with a DNA prime [16]. In addition, NYVAC-SIV induced significantly higher levels of gut-homing Ki67^+^CD4^+^ α_4_β_7_^+^ and Ki67^+^CD38^+^ activated T cells. Both CD4^+^Ki67^+^ subsets correlated with an increased number of transmitted virus variants in vaccinated animals that became infected in a prior study, and the Ki67^+^CD4^+^ α_4_β_7_^+^ T cell subset also correlated with an increased risk of SIV_mac251_ acquisition [15].

Analysis of CCR5 expression in vaccine induced CD4^+^ T-cells did not reveal significant differences between the two groups. Plasmablasts expressing the homing marker CXCR3 for inflammatory sites were increased in the NYVAC-SIV group, and those expressing the α_4_β_7_ homing marker for mucosal sites were decreased as expected, suggesting differential migration to antibody-producing cell tissues. However, we could not detect differences in serum and mucosal binding antibody responses and functional serum antibody responses, such as neutralizing antibodies and ADCC, in the two vaccinated groups. Importantly, we demonstrated that inflammasome activation in classical monocytes is a strong correlate of reduced risk of SIV_mac251_ acquisition, not only following intrarectal exposure to SIV_mac251_ as oberved previously [16], but also following intravaginal exposure to the same virus stock [25]. Collectively, our data on the ALVAC-based vaccine suggest the role of monocyte mediated (*trained*) immunity, an ancient response to pathogens linked to emergency myelopoiesis and durable epigenetic changes in monocytes [61].

**Fig 6:**
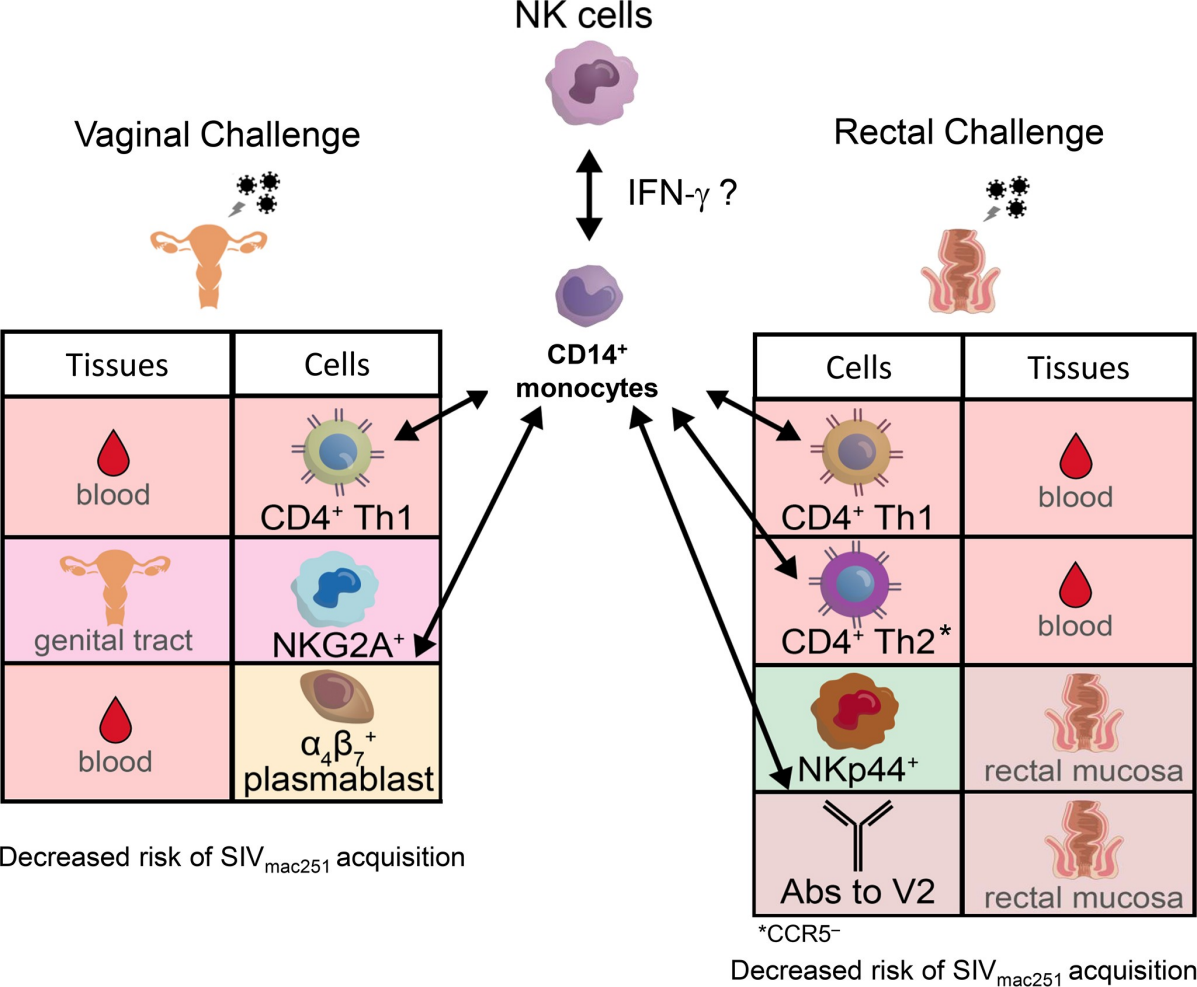
Summary of immune responses that correlated with each other and with virus acquisition. Visual summary of data obtained in the current study (left) and two previously reported independent studies [15, 16]. Vaccine induced cells correlated directly with CD14 monocytes (indicated by arrows) and indirectly with the time of virus acquisition following vaginal (left) and rectal (panel) exposure to the same stock of SIV_mac251_.

Our findings raise the question of how monocytes influence the decreased risk of virus acquisition. We found here an association between vaccine induced CD14^+^ monocytes and vaginal NKG2A^+^ CD107^+^ cells and, in the prior study, between CCL2 (a chemokine produced largely by CD14^+^ classical monocytes) [39] and rectal NKp44^+^ cells [15] (**Fig 6**). Both of these NK subsets correlated with a decreased risk of intravaginal or intrarectal SIV_mac251_ acquisition (**Fig 6**). In addition, we found a trend between blood ADCC activity and ADCC titers with a reduced risk of SIV_mac251_ acquisition in a subgroup of ALVAC-SIV vaccinated macaques, whose plasma was available. NKp44^+^ cells are important for maintaining gut homeostasis [42, 62]. NKG2A^+^ cells express the inhibitory receptor that limits the magnitude and duration of antiviral cytotoxic responses, possibly curbing mucosal tissue inflammation [63]. However, the antibody used to identify the NKG2A^+^ cells population has been shown to cross-react with NKG2C, an activating receptor [64] that precludes, at present, a definitive characterization of these cells. Interestingly, we found in a prior study that mucosal NKG2A^-^NKp44^-^ cells producing IFN-γ were associated with increased SIV_mac251_ acquisition [15]. Further work will thus be necessary to define the role and function of NK cells subsets and monocytes in vaccine protection.

Our data suggest that systemic immunization with the ALVAC-SIV/gp120/alum regimen influences the function of mucosal NK cell responses, likely via monocytes and/or the cytokines and chemokines produced by monocytes. This begs the question of what the functional features of these monocytes are, and whether they are pro-inflammatory or anti-inflammatory.

ALVAC immunization within the first 24 hours of infection increases the production of the pro-inflammatory cytokines IL-1, IL-6, and IL-10 by 20–50-fold. Similarly, alum, another component of the ALVAC-SIV regimen, induces both IL-1β and IL-10 [65].

We therefore favor the hypothesis that monocytes induced by the ALVAC-SIV regimen may be predominantly anti-inflammatory. This hypothesis is supported by the finding that ALVAC is a potent inducer of interferon responses. Recent work in a mouse model study of *T*. *Gondii* infection demonstrated that interferons are cytokines that can “educate” monocytes to became anti-inflammatory [66]. In that study, the production of interferons by bone marrow NK cells during the first few hours of infection was able to prime monocytes to become anti-inflammatory before their egress from bone marrow [66]. Thus, the current and prior studies suggest the hypothesis that the ALVAC-SIV vaccine engages NK cells by inducing an early burst of IFN-γ and infecting CD14^+^ monocytes, activates the inflammasome, and shapes innate responses at mucosal sites associated with a reduced risk of virus acquisition (**Fig 6**). Thus, CD14^+^ monocytes would have an indirect effect on vaccine efficacy by orchestrating other protective responses. Indeed, the frequency of CD14^+^ monocytes correlated with the frequency of vaccine-induced CD4^+^ Th1 in this study, as well as with Th2 negative for CCR5 expression in a prior one where the ALVAC-SIV prime was substituted with a DNA prime [16]. In turn, the number of both CD4^+^ Th1 and Th2 (CCR5^-^) T cell subsets and CD14^+^ monocytes correlated with a reduced risk of virus acquisition (**Fig 5**). The contribution of antibodies to cyclic V2 to the protection observed earlier [15] could not be fully assessed in the current work because of insufficient samples.

In sum, the present study reproduced the efficacy of RV144 in Chinese rhesus macaques following challenge exposure by the vaginal route. The efficacy of the ALVAC-SIV/gp120/alum vaccine regimen is linked to innate responses, such as interferon and myeloid cells during priming, CD4^+^ Th1 responses, NKG2A^+^ cells, and ADCC. This work thus defines novel correlates of risk and demonstrates the reproducibility of the efficacy observed in the ALVAC-based regimen in the rigorous SIV_mac251_ macaque model, thereby establishing a benchmark for the future improvement of HIV vaccine candidates.

## Methods

### Animals, vaccines and SIV_mac251_ challenge

All animals included this study were female rhesus macaques (*Macaca mulatta*) imported from China and obtained from the Washington National Primate Research Center (Seattle, WA). Animals were not tested for MHC-I expression. The care and use of the animals were in compliance with all relevant NIH institutional guidelines. A total of 65 female rhesus macaques were randomized into five groups (the five groups are referred to in the text as ALVAC-SIV/gp120 [ALVAC-SIV], NYVAC-SIV/gp120 [NYVAC-SIV], ALVAC-control, NYVAC-control, and naïve). The animals in the ALVAC and NYVAC-SIV groups were immunized at weeks 0, 4, 12, and 24 with intramuscular inoculations at 10^8^ PFU of either ALVAC-SIV (vCP180) or NYVAC-SIV (VP1071) carrying the identical Env-Gag-Pol genes from SIV_K6W_ [67]. At weeks 12 and 24, the animals from these groups received 200 μg of native SIV_mac251_ gp120 protein [12] formulated with 5mg of alum, as a boost in the opposite thigh of the vector immunization.

The control groups included 10 animals each, which received either parental ALVAC-SIV or NYVAC-SIV vectors and alum. Five naïve control animals were included before the challenge phase. Animals were challenged four weeks after the last immunization (week 28) with SIV_mac251_ [25] at 120 TCID_50_ for each challenge as previously described [15, 68]. Animals that tested negative for SIV-RNA in plasma were rechallenged with up to a maximum of 12 weekly administrations.

Given the large number of macaques, the study was split into two parts. Animals from Part 1 (n = 24; 8 ALVAC-SIV/gp120; 8 NYVAC-SIV/gp120; 4 ALVAC-control; 4 NYVAC-control) were housed, immunized and challenged at Washington National Primate Research Center and animals from Part 2 (n = 41; 12 ALVAC-SIV/gp120; 12 NYVAC-SIV/gp120; 6 ALVAC-control; 6 NYVAC-control; 5 naïve) were housed, immunized, and challenged at Advanced Bioscience Laboratories (ABL, Inc., Rockville, MD).

### Measurement of viral RNA and DNA

Plasma SIV_mac251_ RNA levels were quantified by nucleic acid sequence-based amplification [69]. SIV/DNA levels in mucosal biopsies from week 2 post-infection were quantified by a real-time qPCR with sensitivity set at ten copies x 10^6^ cells, as previously described [70].

### IFN-γ ELISpot

IFN-γ production by CD4^+^ T cells or CD8^+^ T cells was assessed through ELISpot assays, as a reaction to the optimal CTL epitope peptide P18 [71] or a pool of 47, 15 aa-long overlapping HIV-1 IIIB Env gp120 peptides (Centralized Facility for AIDS Reagents, Potters Bar, U.K.). Multiple 96-well multiscreen plates (Millipore, Bedford, MA) were coated overnight with 10 μg/ml rat anti-mouse IFN-γ (BD PharMingen, San Diego, CA) in PBS (100 μl/well), then washed with 0.25% Tween 20 endotoxin-free Dulbecco’s PBS (Life Technologies, Gaithersburg, MD). The reaction was blocked for 2 hours at 37°C with PBS containing 5% FBS. After washing the plates three times with 0.25% Tween 20 Dulbecco’s PBS, they were rinsed with 10% FBS-RPMI 1640, and incubated in triplicate with 5 × 10^5^ PBMCs/well in a 100-μl reaction volume with peptide at a concentration of 8 μg/ml. After an 18-hour incubation, Dulbecco’s PBS containing 0.25% Tween 20 was used to wash the plates five times, and once with distilled water. After a 16-hour incubation with 75 μl/well 5 μg/ml biotinylated rat anti-mouse IFN-γ, the plates were washed six times with Coulter wash (Coulter, Miami, FL), and incubated with a 1/500 dilution of streptavidin-AP (Southern Biotechnology Associates, Birmingham, AL) for 2.5 hours. Later, the plates were washed five times with Coulter wash and once with PBS, then developed with nitro blue tetrazolium/5-bromo-4-chloro-3-indolyl phosphate chromogen (Pierce, Rockford, IL) and stopped by washing with tap water. Lastly, the plates were air-dried and read using an ELISpot reader (Hitech Instruments, Edgemont, PA).

### Neutralizing antibodies

Serum neutralizing activity was measured as reduction in expression of luciferase reporter gene after a single round of infection in TZM-b1 cells, as described previously [72]. TZM-b1 cells were obtained from the NIH AIDS Research and Reference Reagent Program as contributed by J. Kappes and X. Wu. Briefly, 200 TCID_50_ of pseudoviruses were incubated with serial 3-fold dilutions of test sample in duplicate, in a final volume of 150 μl for 1 out of 18 h at 37°C in 96-well flat-bottom culture plates. Freshly trypsinized cells (10,000 cells in 100 μl of growth medium containing 75 μg ml^−1^ DEAE-dextran) were then added to each well. Two sets of control wells were included as controls: the one set to receive cells and virus (positive control) and the other set to receive cells only (background control). For measurement of luminescence, cells were then transferred to 96-well black solid plates (Costar) and signal was detected using the Britelite luminescence reporter gene assay system (PerkinElmer Life Sciences). Neutralization titers were defined as the dilution at which relative luminescence units were reduced by 50% compared to that in positive control wells minus the background signal detected in negative control wells. Stocks of Env-pseudo-typed viruses (SIV_mac251.6_ and SIV_mac251.30_) were prepared by transfection in 293T cells and titrated in TZM-bl cells, as previously described [73].

### SIV Env-specific serum IgG binding antibody assay

The total macaque IgG were measured by macaque IgG ELISA and a custom SIV bAb multiplex assay (SIV-BAMA) was used to quantify SIV Env-specific IgG antibodies in serum as previously described [74, 75]. Purified IgG (DBM5) from a SIV-infected macaque (kindly provided by M. Roederer, VRC, NIH) was used as the positive control to calculate SIV antibody concentration. A Levy-Jennings Plot was used to track positive controls for each group. Specific activity was calculated from the total macaque IgG levels and the SIV specific concentrations. The quantitation of antibodies against native V1/V2 epitopes was performed through binding assays against native SIV V1/V2 antigens expressed as gp70-fusion proteins related to the CaseA2 antigen used in the RV144 correlate study (provided by A. Pinter). These synthetic proteins contain the glycosylated, disulfide-bonded V1/V2 regions of SIV_mac239_, SIV_mac251_, and SIVs_mE660_ (corresponding to AA 120–204 of HXB2 Env), linked to residue 263 of the SU (gp70) protein of Fr-MuLV.

### ADCC assay

ADCC activity was assessed as previously described using a constitutive GFP expressing EGFP-CEM-NKr-CCR5-SNAP cells as target [43]. Briefly, one million target cells were incubated with 50 μg of SIV gp120 wild type protein for 2 h at 37°C, washed, and labeled with SNAP-Surface Alexa Fluor 647 (New England Biolabs, Ipswich, MA; cat.# S9136S) as recommended by the manufacturer for 30 min at RT. Heat inactivated plasma samples were serially diluted (7 ten-fold dilutions starting at 1:10) and 100 μl were added to a 96-well V-bottom plate (Millipore Sigma). Following this, 5000 target cells (50 μl) and 250,000 human PBMCs (50 μl) as effectors were added to each well to give an effector/target (E/T) ratio of 50:1. The plate was incubated at 37°C for 2 h followed by two PBS washes. The cells were re-suspended in 200 μl of a 2% PBS–paraformaldehyde solution and acquired on an LSRII equipped with a high throughput system (BD Biosciences, San Jose, CA). Specific killing was measured by loss of GFP from the SNAP-Alexa647^+^ target cells. Target and effector cells cultured in the presence of medium were used as negative controls. Anti-SIVmac gp120 monoclonal antibody, KK17 (NIH AIDS reagent program), was used as a positive control. Normalized percent killing was calculated as the following: (killing in the presence of rectal secretion—background)/ (killing in the presence of KK17- background) ×100. The ADCC endpoint titer is defined as the reciprocal dilution at which the percent ADCC killing was greater than the mean percent killing of the negative control wells containing medium, target and effector cells, plus three standard deviations.

### IgG linear epitope mapping in serum

The first week after the last immunization, 1:20-diluted sera were added to ELISA plates coated with overlapping peptides encompassing the entire SIV_K6W_ gp120 amino acid sequence [76]. Linear peptide mapping of serum was performed by microarray (PepStar) [77]. Briefly, JPT Peptide Technologies GmbH (Germany) produced array slides designed by Dr. B. Korber (Los Alamos National Laboratory) by printing a library of overlapping peptides (15-mers overlapping by 12) covering full-length gp160 of SIV_mac239_ and SIV_smE660_ onto epoxy glass slides (PolyAn GmbH, Germany). One printing area of each quad-slide contained three identical sub-arrays, each containing the full peptide library. After hybridizing the slides using a Tecan HS4000 Hybridization Workstation, the samples were incubated with DyLight 649-conjugated goat anti-rabbit IgG (Jackson ImmunoResearch, PA). Later, fluorescence intensity was measured with a GenePix 4300 scanner (Molecular Devices) and analyzed with the GenePix software. The background value was subtracted from the binding intensity of the post-immunization serum to each peptide, defined as the median signal of the pre-bleed serum for that peptide plus three-times the standard error among the three sub-arrays on slide. The total IgG concentration measured was used to normalize the values for each peptide as reported above (Unit = signal intensity/μg/ml total IgG).

### Plasmablast staining in peripheral blood

We measured the frequency of plasmablasts in the peripheral blood of twenty macaques vaccinated with ALVAC-SIV/gp120 and twenty macaques vaccinated with NYVAC-SIV/gp120 before vaccination and at week 25 (7 days after the last immunization). To stain the cells, the following markers were labeled: CD3 (SP34^-^2), CD14 (M5E2), CD16 (3G8), and CD56 (B159), all in ALEXAFluor700 (BD Biosciences); CD19^-^ PE^-^Cy5 (J3-119, Beckman Coulter), CD20^-^ Qdot650 (2H7, eBiosciences), CD38^-^ FITC (Clone AT-1, StemCell), CD39^-^ BV421 (MOCP-21, BioLegend), Ki67^-^ PE (B56, BD Biosciences), and CD183^-^PE^-^CF594 (CXCR3; 1C6 BD, Biosciences). Dr. A. A. Ansari kindly provided the anti-α4β7 (Act-1) reagent (cat. #11718) through the NIH AIDS (NIAD) Reagent Program, Division of AIDS. Cytofix/Cytoperm (BD Biosciences) was used to allow intracellular staining. LSR II (BD Biosciences) was used to evaluate acquisition and the resulting data were analyzed with FlowJo (TreeStar). Gating of lineage negative (CD3^-^ CD14^-^ CD16^-^ CD56^-^) CD20^+^ CD21^-^ Ki67^+^ CD38^+^ CD39^+^ was used to identify plasmablasts [78]. The expression of CXCR3 or α4β7 on the plasmablasts was used to calculate the frequency of the CXCR3^+^ and α4β7^+^ plasmablasts.

### Monocyte staining in blood

To allow identification of monocytic myeloid cells, PBMCs (5–10 × 106 cells) were stained with PE- Cy7 anti-CD20 (2H7; 560735, BD Biosciences), PE-Cy7 anti-CD3 (SP34-2; 563916, BD Biosciences), APC anti-CD14 (M5E2; 561390, BD Biosciences), BV786 anti-NHP-CD45 (D058-1283; 563861, BD Biosciences), HLA-DR-APC-Cy7 (L243; 307618, BioLegend), BV421 anti-CD192 (CCR2) (48607; 564067, BD Biosciences), FITC anti-CD16 (3G8; 555406, BD Biosciences) and PE-CF594 anti-CD184 (CXCR4) (12G5; 562389, BD Biosciences), as well as Aqua LIVE/DEAD kit (L34966, Invitrogen) to dismiss dead cells. CD45^+^Lin^−^ (CD3 and CD20) was considered as a signature of myeloid cell populations. Monocyte populations were further recognized and sub-categorized according to the expression of CD14 and CD16, where classical monocytes were identified as Lin^−^CD45^+^CD14^+^CD16^−^HLA-DR^+^, intermediate as Lin^−^CD45^+^CD14^+^CD16^+^HLA-DR^+^ and non-classical as Lin^−^CD45^+^CD14^−^CD16-HLA-DR^+^. Data are shown as frequency of CD45 cells or as frequency of HLA-DR cells. Acquisition was then done on an LSRII (BD Biosciences), and marker expression was examined in real-time using the software FACSDiva (BD Biosciences). Data were analysed more in detail with FlowJo version 10.1 (TreeStar, Inc.).

### Staining for vaginal mucosa NK/ILCs

Pinch biopsy specimens obtained from the cervix and vaginal tract were washed and incubated for 1 h with collagenase D at a concentration of 2 mg/ml in Iscove’s medium with antibiotics and amphotericin. Following incubation, the remaining tissue was mechanically disrupted to obtain a mononuclear cell suspension as described previously [62, 79]. Filtered single-cell suspensions of mononuclear cells use for used in an intracellular cytokine assay performed as previously described. Cells were stimulated with Phorbol Myristate Acetate (PMA) (50 ng/mL) and ionomycin (1 μg/mL), or were left unstimulated in the presence of Golgi Plug^TM^ (Brefeldin A) and Golgi Stop^TM^ (Monensin), Anti-CD107a (eBioH4A3) (eBiosciences). Samples were then cultured at 37°C in 5% CO2 for 12h. Negative controls were represented by unstimulated (medium alone) samples. After incubation, cells were washed and stained for surface markers with following anti-human fluorochrome-conjugated mAbs, which are described to cross-react with rhesus macaques. PE-Cy7 anti-CD56 (NCAM 16.2), Alexa Fluor 700 anti-CD3 (SP34-2), Allophycocyanin-Cy7 anti-CD3 (SP34-2), and Alexa Fluor 700 anti-Ki67 (B56; all from BD Biosciences, San Jose, CA); eFluor 605NC anti-CD20 (2H7), and eFluor 605NC anti-CD8a (RPA-T8; all from eBioscience, San Diego, CA); PE anti-NKG2A (Z199), ECD anti-CD16 (3G8), PE-Cy5 anti-NKp46 (BAB281; Beckman Coulter, Fullerton, CA), allophyco-cyanin anti-NKp44 (P44-8) and Dead cells were excluded through yellow and aqua LIVE/DEAD viability dyes (Invitrogen). After incubation for 30 mins, cells were washed and permeabilized using Cytofix/Cytoperm (BD Biosciences). Then, intracellular staining was performed using following antibodies. V450 anti-IFN-γ (B27), Alexa 488 anti-IL17 (eBio64DEC17), PerCP/Cy5.5 anti-TNF-a (Mab11; BioLegend, San Diego, CA). At least 100,000 singlet events (mononuclear cells) were acquired on an LSR II (BD Biosciences). FlowJo Software (TreeStar) was used to analyze the data.

### CD4^+^ T cell staining

Cells were stained as described elsewhere [15]. CD4^+^ T cells were analyzed in blood at week 26. PBMCs were stained with PerCPCy5.5 anti-CD4 (L200; cat. #552838, 5 μl), AlexaFluor 700 anti-CD3 (SP34-2, cat. #557917, 0.2 mg/ml), and BV650 anti-CCR5 (3A9, 5 μl), PeCy5 anti-CD95 (DX2, #559773 5 μl; BD Biosciences, San Jose, CA), PE-eFluor 610 anti-CD185 (CXCR5; MU5UBEE, **#**61-9185-42 5 μl; eBiocence, FITC anti-Ki67 cat # 71-5776-40 μl), and CD-38-PE and APC anti-α4β7, provided by the NIH Nonhuman Primate Reagent Resource (R24 OD010976; NIAID contract HHSN272201300031C). Gating was performed on live CD3^+^CD4^+^ cells and on vaccine induced Ki67^+^ cells.

### RNA isolation and microarray processing

Total RNA was isolated from Paxgene tubes by the use of Paxgene Blood RNeasy minikits (Qiagen) following the manufacturer's protocol. RNA quality was assessed on an Agilent 2100 bioanalyzer, using the nanochip format, and only intact RNA was used for microarray analysis. Fifty nanograms of each RNA sample were hybridized to one Agilent 8×60 rhesus macaque array (Agilent, G4102A). Raw array intensities were read, background corrected with the norm-exp method, quantile-normalized and log2 transformed for variance stability, using functions implemented in the R package *limma*. For each array, intensities of replicated probes were averaged. Two arrays (for samples P168_R808_NYVAC-SIV_w4_0h and P168_A06028_ALVAC-SIV_w0_6h) with low overall raw intensities were considered outliers and excluded from downstream analysis. Technical replicates arrays (2 replicates for 4 samples) were averaged prior to the differential gene-expression analysis. To identify genes differentially expressed between ALVAC-SIV/gp120 and NYVAC-SIV/gp120 and their respective empty vector control (ALVAC-control and NYVAC-control) adjusting for pre-vaccination transcriptomic profile, we first subtracted the pre-vaccination expression from all post-vaccination samples separately for each donor (*i*.*e*. to obtain fold-change post/pre-vaccination) and fitted a linear model with the treatment as independent variable for each gene. A moderated *t*-test, as implemented in the R package *limma*, was used to assess the statistical significance of the difference between vaccine and empty vector control. Benjamini & Hochberg correction was used to adjust for multiple testing.

Gene Set Enrichment Analysis (GSEA) was used to identify pathways that were enriched in the set of genes that distinguished the treatment groups, where one treatment group (ALVAC-SIV) showed a reduced rate of SIV_mac251_ acquisition. In GSEA, the most varying probes across samples was used to remove redundant probes annotated to a same gene. The gene list ranked by LIMMA moderated t-statistic were used as input for the GSEA analysis. The pathways (*i*.*e*. genesets) database used for all GSEA analysis were the Molecular Signatures Database (version 6.1) genesets and blood cells markers^48^. The GSEA Java desktop program was downloaded from http://www.broadinstitute.org/gsea/index.jsp and the default parameters of GSEA preranked module (number of permutations: 1000; enrichment statistic: weighted; seed for permutation: 101, 15 ≤ gene set size ≤ 500) were applied for analyses.

### Statistical analysis

Continuous factors between the two groups were compared using the Wilcoxon rank-sum test. The Spearman’s rank correlation was used to perform correlation analyses with the calculation of exact permutation p values. The Log-rank test of the discrete-time proportional hazards model defined the number of challenges before acquisition of infection. The changes in plasmablast levels from pre- to post-vaccination were evaluated by the paired Wilcoxon signed-rank test.

### Code availabilities

Code used to generate the figures is available at https://github.com/sekalylab/p168

### Ethics Statement

All animals included in this study were female rhesus macaques (*Macaca mulatta*) imported from China and obtained from the Washington National Primate Research Center (Seattle, WA). The care and use of the animals were in compliance with all relevant NIH institutional guidelines. Animals were cared for in accordance with the American Association for the Accreditation of Laboratory Animal Care (AAALAC) standards in AAALAC-accredited facilities (Animal Welfare Assurance A4149-01). All animal care and procedures were carried out under protocols approved by the NCI or NIAID Animal Care and Use Committees (ACUC; Protocol P168) and/or the University of Washington Institutional Animal Care and Use Committee (UW protocol #4266–04). The studies adhered to the regulations/guidelines as outlined in the Guide for the Care and Use of Laboratory Animals, Eighth Edition; The Animal Welfare Act; CDC “Biosafety in Microbiological and Biomedical Laboratories”; and Public Health Service Policy on Humane Care and Use of Laboratory Animals. The Animal Care and Use Committee approved all experiments and met all applicable federal and institutional standards. Animals were closely monitored daily for any signs of illness, and appropriate medical care was provided as needed. Animals were housed individually during the challenge phase to reduce the risk of transmission of SIV or other viruses. All clinical procedures, including biopsy collection, administration of anesthesia and analgesics, and euthanasia, were carried out under the direction of a laboratory animal veterinarian. Steps were taken to ensure the welfare of the animals and minimize discomfort of all animals used in this study. Animals were fed daily with a fresh diet of primate biscuits, fruit, peanuts, and other food items to maintain body weight or normal growth. Animals were monitored for mental health and provided with physical enrichment including sanitized toys, destructible environments (cardboard and other paper products), and audio stimulation.

## Supporting information

S1 Fig Acquisition of SIV_mac251_ in control groups and monocyte subset frequency in blood. (A) Acquisition curves of the three control groups immunized with parental ALVAC (10 animals) or parental NYVAC (9 animals), or left naïve (5 animals). The null hypothesis of equal survival distributions in the three control groups is not rejected by the Log Rank test, allowing the groups to be combined. Monocyte subsets obtained at week 26 (two weeks after the last immunization) were measured in animals immunized with ALVAC-SIV (n = 10) or NYVAC-SIV (n = 11) and naïve animals (n = 7; 4 ALVAC-SIV, 1 NYVAC-SIV, 2 ALVAC-control, sampled at week 4). (B)
Total monocytes at week 26 (two weeks after the last immunization) in both animal groups. Frequency of (C) CD14^+^CD16^-^ and (D) CXCR4^+^ classical monocytes, (E) CD14^+^CD16^+^ and (F) CXCR4^+^ intermediate monocytes, and (G) CD14^-^CD16^+^ and (H) CXCR4^+^ non classical monocytes. Percentage of CCR2^+^ (I) classical, (J) intermediate, or (K) non-classical monocytes. (L) Correlation of non-classical monocytes and (M) CD14^+^HLA^-^DR^-^ (MDSC) with the number of intravaginal challenge necessary to acquire SIV_mac251._
(PPTX)

S2 Fig SIV-specific T cells and antibodies. (A) Representative flow cytometric plots defining NK/ILCs in the vaginal mucosa of rhesus macaques. NK/ILCs were identified using a side-scatter versus forward-scatter gate and phenotypically defined as CD3^−^CD20^−^ and NKG2A^+^, NKp44^+^ cells, or as NKG2A^−^NKp44^−^ cells. Comparison of percent (B) ADCC killing and (C) ADCC titer in 7 ALVAC-vaccinated and 7 NYVAC-vaccinated macaques one week following the final immunization (week 25). Horizontal lines represent the median. (D) Gag and (E) Envelope specific ELISpot in PBMCs of vaccinated animals over time. ALVAC-SIV = 18 animals; NYVAC-SIV = 20 animals. Arrows indicate the time of immunization according to the regimen presented in Fig 1A. (F) Representative plot of the T cell assay in the blood of two animals from the NYVAC-SIV and two animals from the ALVAC-SIV groups. Increased frequencies of Th2 and Th1 CD4^+^ T cells were observed in NYVAC-SIV and ALVAC-SIV, respectively. (G) Percentage of circulating Ki67^+^ CD95^+^ CD4^+^ T cells expressing CCR5 in 6 animals in the ALVAC group and 8 animals in the NYVAC group (week 26).
(PPTX)

S3 Fig Study design and microarray analysis sampling timepoints. (A) Logarithmic mean ± s.d. of SIV/gp120-specific serum antibody titers in the ALVAC-SIV (n = 18), NYVAC-SIV (n = 20), and pooled Control groups (n = 19). Arrows represent the time of immunization. (B) Vaginal IgG to the SIV_mac251_ gp70 V1/V2 scaffold at week 26. (C-D) Titers of neutralizing antibodies to (C) Tier 1A SIV_mac251.6_ and (D) Tier 2 SIV_mac251.30_. (E) Timepoints of the transcriptomic analysis. (F) Heatmap of all genes differentially expressed between ALVAC-SIV vs. ALVAC-Control, and NYVAC-SIV vs. NYVAC-Control (LIMMA: adj. p-value ≤ 0.05). A blue-to-red color gradient represents the log2 fold-change between the vaccine groups.
(PPTX)

S4 Fig Interferon genes associated with SIV challenges to infection. Heatmap of interferon geneset associated with the number of SIV challenges to infection in at least one vaccine/immunization/timepoint. GSEA was used to assess the enrichment of the 31 interferon genesets in the MSigDB databases. The Normalized Enrichment Score (NES) of the genesets is depicted in the heatmap with a blue-white-red color gradient; NES < 0 indicates that the geneset is associated with increased risk of acquisition, while NES > 0 means that the interferon geneset is associated with lower risk of acquisition (i.e. protection). Enrichments associated with FDR > 0.05 are shown in grey. The x axis records the number of weeks and hours from vaccination (eg., “w12.24” = 12 weeks, 24 h post-vaccination).
(PPTX)
