# Supplementary figures and images for: Engagement of monocytes, NK cells, and CD4+ Th1 cells by ALVAC-SIV vaccination results in a decreased risk of SIVmac251 vaginal acquisition
Source: PLoS Pathog. 2020 Mar 12;16(3):e1008377. doi: 10.1371/journal.ppat.1008377 (PMC7093029; doi:10.1371/journal.ppat.1008377)

## Slide 1
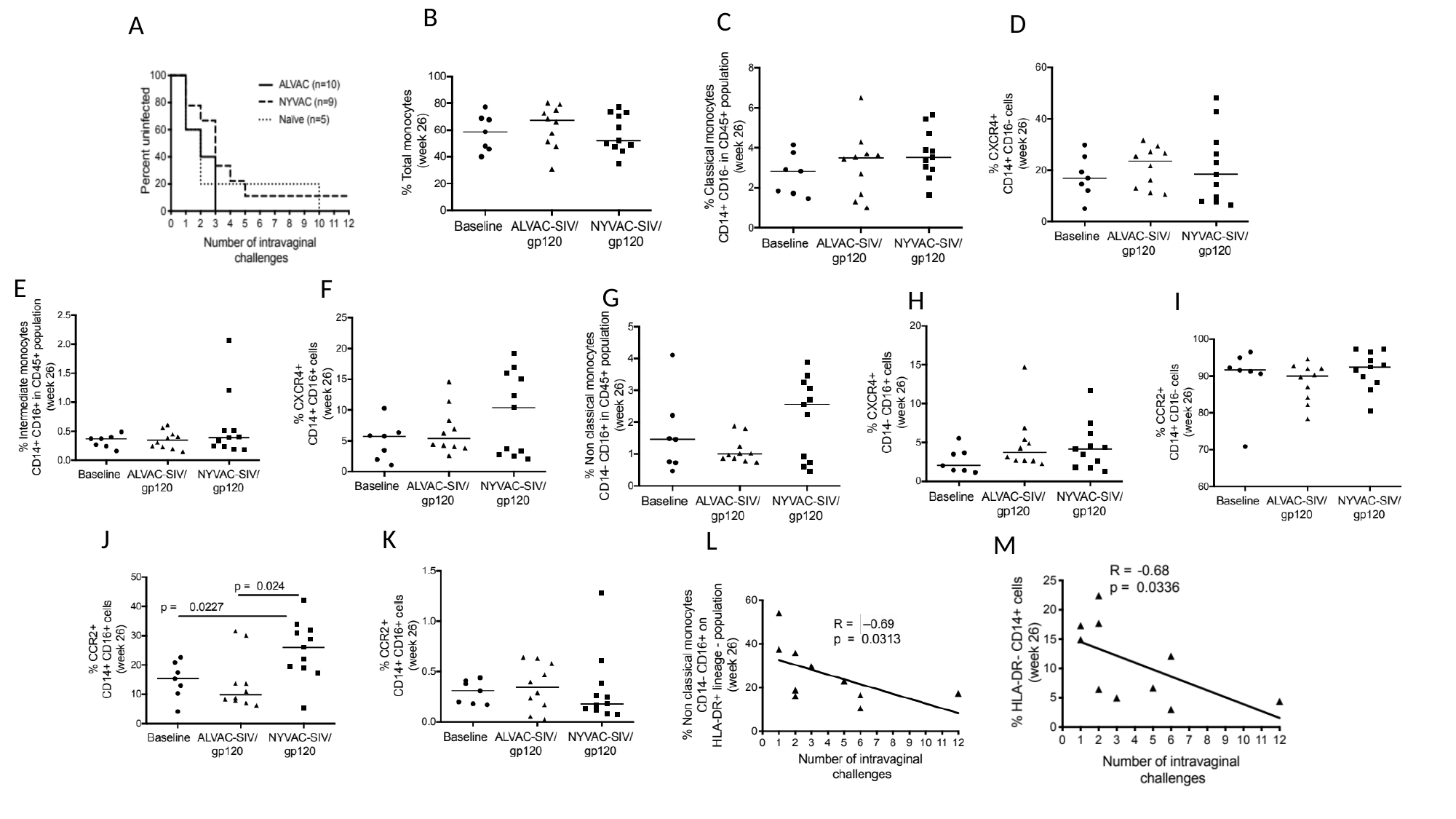

B
C
D
A
E
F
G
I
H
K
L
J
M

Supplement: S1 Fig — (A) Acquisition curves of the three control groups immunized with parental ALVAC (10 animals) or parental NYVAC (9 animals), or left naïve (5 animals). The null hypothesis of equal survival distributions in the three control groups is not rejected by the Log Rank test, allowing the groups to be combined. Monocyte subsets obtained at week 26 (two weeks after the last immunization) were measured in animals immunized with ALVAC-SIV (n = 10) or NYVAC-SIV (n = 11) and naïve animals (n = 7; 4 ALVAC-SIV, 1 NYVAC-SIV, 2 ALVAC-control, sampled at week 4). (B) Total monocytes at week 26 (two weeks after the last immunization) in both animal groups. Frequency of (C) CD14+CD16- and (D) CXCR4+ classical monocytes, (E) CD14+CD16+ and (F) CXCR4+ intermediate monocytes, and (G) CD14-CD16+ and (H) CXCR4+ non classical monocytes. Percentage of CCR2+ (I) classical, (J) intermediate, or (K) non-classical monocytes. (L) Correlation of non-classical monocytes and (M) CD14+HLA-DR- (MDSC) with the number of intravaginal challenge necessary to acquire SIVmac251. (PPTX) [file ppat.1008377.s001.pptx]

## Slide 1
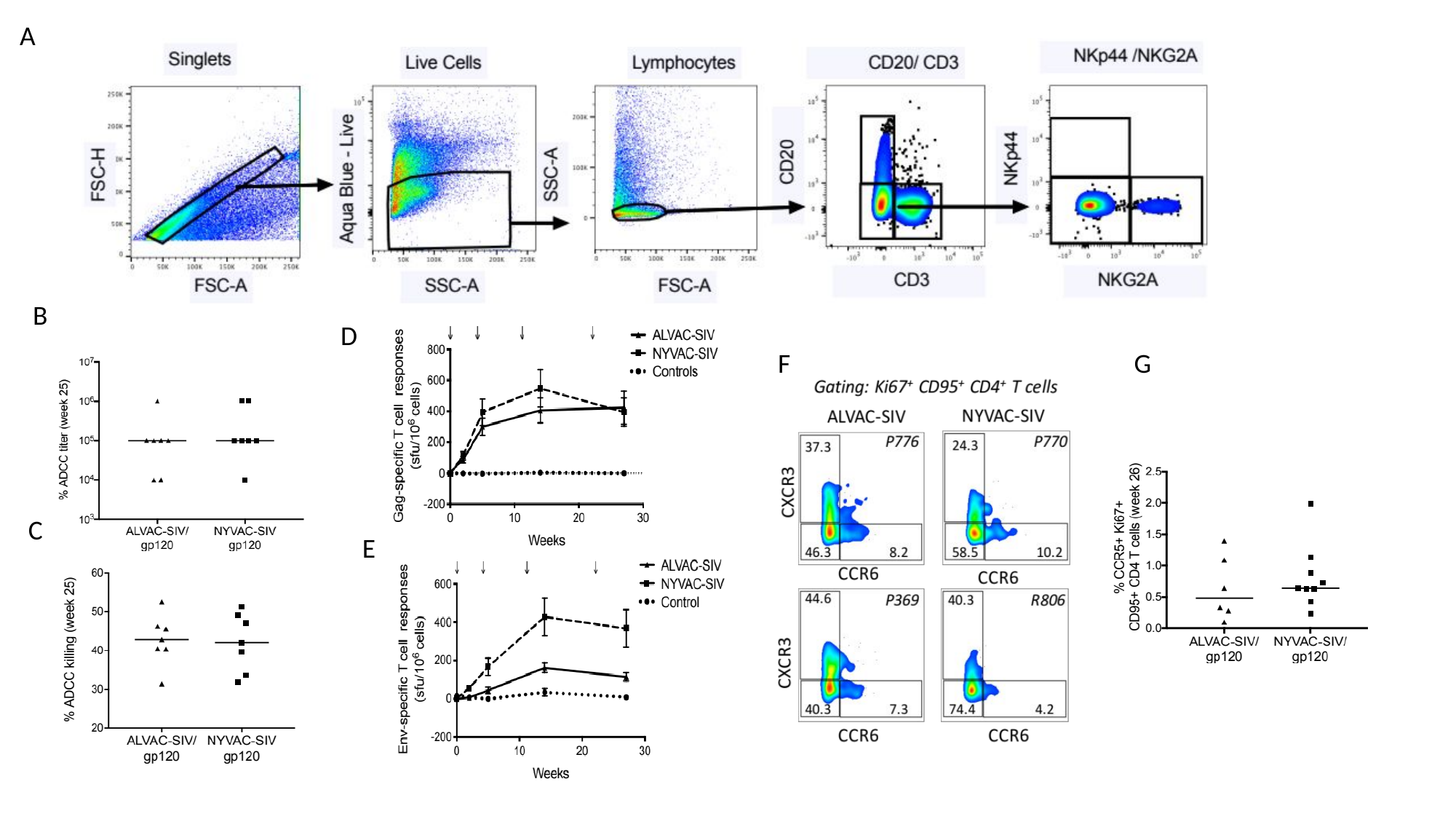

A
B
D
F
G
C
E

Supplement: S2 Fig — (A) Representative flow cytometric plots defining NK/ILCs in the vaginal mucosa of rhesus macaques. NK/ILCs were identified using a side-scatter versus forward-scatter gate and phenotypically defined as CD3−CD20− and NKG2A+, NKp44+ cells, or as NKG2A−NKp44− cells. Comparison of percent (B) ADCC killing and (C) ADCC titer in 7 ALVAC-vaccinated and 7 NYVAC-vaccinated macaques one week following the final immunization (week 25). Horizontal lines represent the median. (D) Gag and (E) Envelope specific ELISpot in PBMCs of vaccinated animals over time. ALVAC-SIV = 18 animals; NYVAC-SIV = 20 animals. Arrows indicate the time of immunization according to the regimen presented in Fig 1A. (F) Representative plot of the T cell assay in the blood of two animals from the NYVAC-SIV and two animals from the ALVAC-SIV groups. Increased frequencies of Th2 and Th1 CD4+ T cells were observed in NYVAC-SIV and ALVAC-SIV, respectively. (G) Percentage of circulating Ki67+ CD95+ CD4+ T cells expressing CCR5 in 6 animals in the ALVAC group and 8 animals in the NYVAC group (week 26). (PPTX) [file ppat.1008377.s002.pptx]

## Slide 1
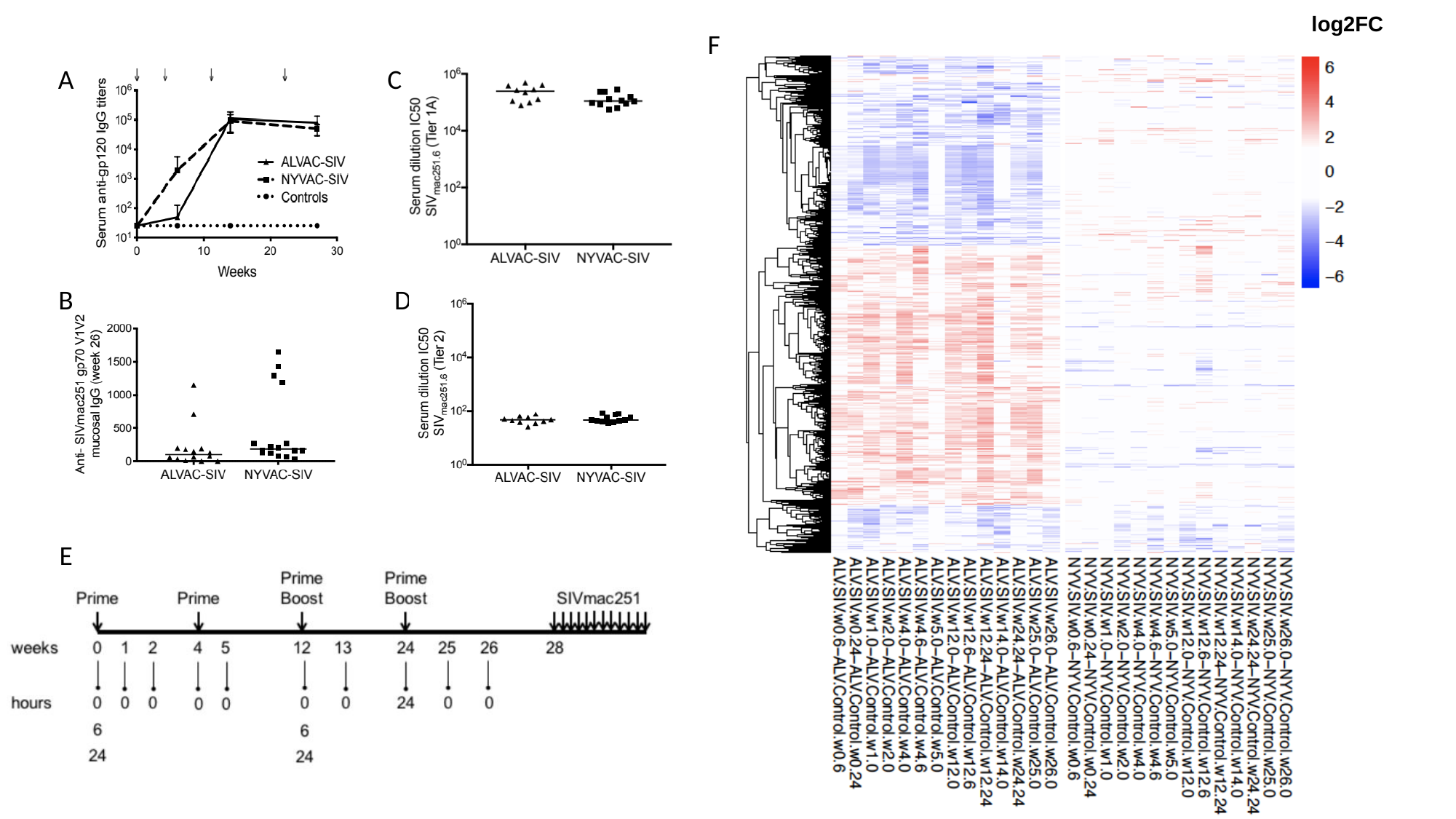

log2FC
F
A
C
B
D
E

Supplement: S3 Fig — (A) Logarithmic mean ± s.d. of SIV/gp120-specific serum antibody titers in the ALVAC-SIV (n = 18), NYVAC-SIV (n = 20), and pooled Control groups (n = 19). Arrows represent the time of immunization. (B) Vaginal IgG to the SIVmac251 gp70 V1/V2 scaffold at week 26. (C-D) Titers of neutralizing antibodies to (C) Tier 1A SIVmac251.6 and (D) Tier 2 SIVmac251.30. (E) Timepoints of the transcriptomic analysis. (F) Heatmap of all genes differentially expressed between ALVAC-SIV vs. ALVAC-Control, and NYVAC-SIV vs. NYVAC-Control (LIMMA: adj. p-value ≤ 0.05). A blue-to-red color gradient represents the log2 fold-change between the vaccine groups. (PPTX) [file ppat.1008377.s003.pptx]

## Slide 1
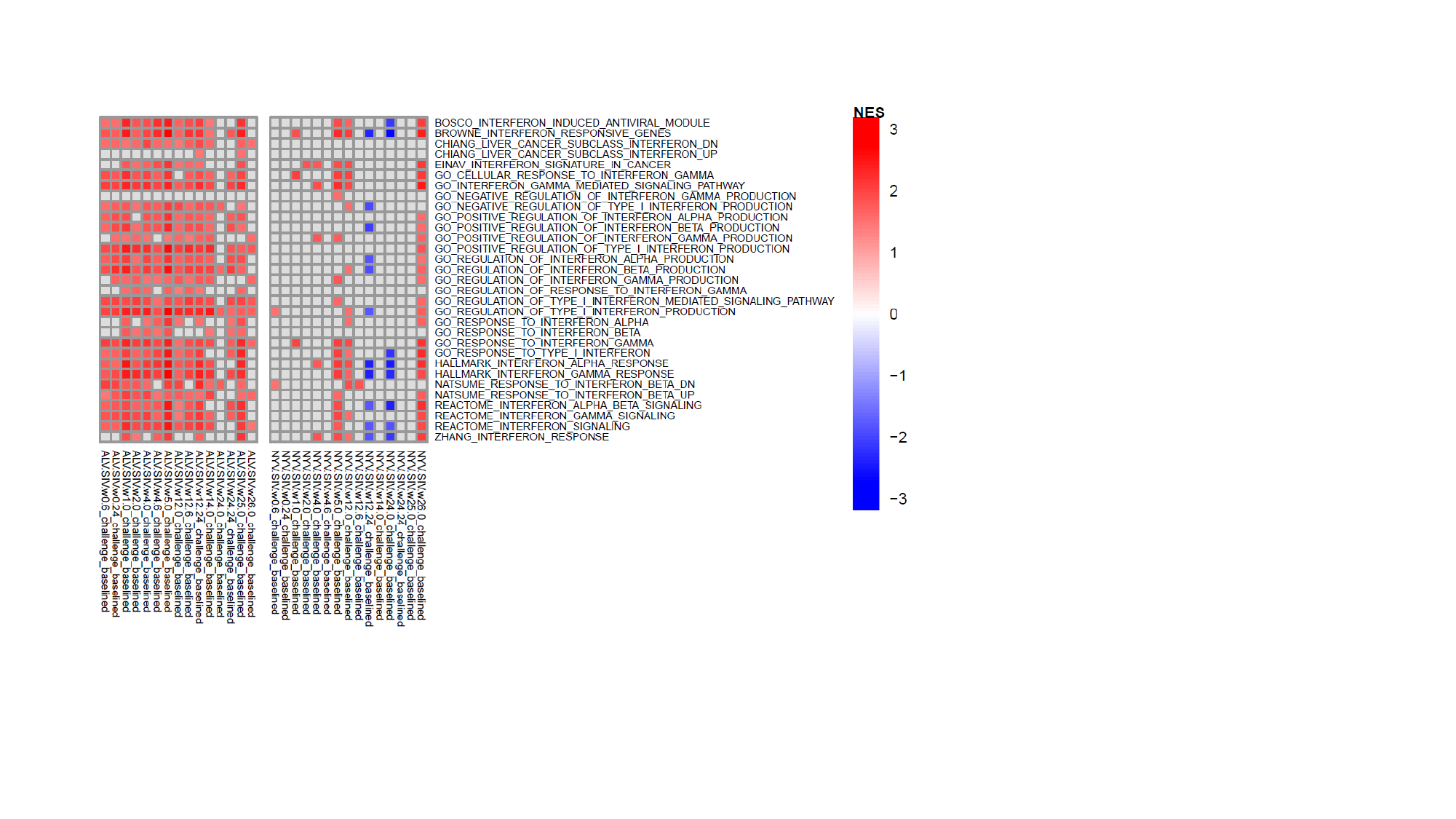

Supplement: S4 Fig — Heatmap of interferon geneset associated with the number of SIV challenges to infection in at least one vaccine/immunization/timepoint. GSEA was used to assess the enrichment of the 31 interferon genesets in the MSigDB databases. The Normalized Enrichment Score (NES) of the genesets is depicted in the heatmap with a blue-white-red color gradient; NES < 0 indicates that the geneset is associated with increased risk of acquisition, while NES > 0 means that the interferon geneset is associated with lower risk of acquisition (i.e. protection). Enrichments associated with FDR > 0.05 are shown in grey. The x axis records the number of weeks and hours from vaccination (eg., “w12.24” = 12 weeks, 24 h post-vaccination). (PPTX) [file ppat.1008377.s004.pptx]
